# Effects of neuromuscular-cognitive training on ACL injury risk factors in female volleyball athletes with dynamic knee valgus: Protocol for a randomized controlled trial

**DOI:** 10.1371/journal.pone.0353944

**Published:** 2026-08-25

**Authors:** Farzaneh Ramezani, Farzaneh Saki, Mohammad Reza Zoghi Paydar

**Affiliations:** 1 Department of Exercise Rehabilitation, Faculty of Sports Science, Bu-Ali Sina University, Hamedan, Iran; 2 Department of Psychology, Faculty of Economic and Social Sciences, Bu-Ali Sina University, Hamedan, Iran; Drexel University School of Biomedical Engineering Science and Health Systems, UNITED STATES OF AMERICA

## Abstract

**Background:**

Female volleyball athletes are at high risk of non-contact anterior cruciate ligament (ACL) injuries, particularly during high-impact activities such as single-leg landings after a spike or rapid directional changes. These movements often occur under cognitively demanding conditions, including split attention, fast decision-making, and responses to unpredictable stimuli, which can impair neuromuscular coordination and exacerbate motor deficits, such as dynamic knee valgus (DKV). Although neuromuscular training programs exist, many have not been specifically tailored for female volleyball athletes or integrated cognitive demands mimicking real-game scenarios.

**Objective:**

This study protocol describes a planned randomized controlled trial designed to evaluate the effects of an eight-week neuromuscular-cognitive training protocol in reducing risk factors for ACL injury among female volleyball players with DKV.

**Methods:**

This study protocol describes a two-arm, parallel-group randomized controlled trial that will assess an eight-week neuromuscular-cognitive training program for female volleyball players aged 18–23 with clinically confirmed DKV and competitive experience. Participants will be randomly assigned to either an experimental group that will receive integrated neuromuscular-cognitive training or a control group that will receive standard neuromuscular training without cognitive components. The intervention will combine sport-specific motor tasks with cognitive challenges aimed at improving neuromuscular control and decision-making during dynamic movements. The primary outcome will be single-leg landing kinematics under cognitive and non-cognitive conditions. Secondary outcomes will include cortical brain activity, lower limb muscle activation, single-leg balance, muscle strength, and knee joint proprioception. Additional measures will involve participant feedback on training intensity and satisfaction.

**Discussion:**

If effective, this protocol will provide evidence for integrating cognitive training into ACL injury prevention programs for female volleyball athletes. By examining the interaction between cognitive load and neuromuscular control under sport-specific conditions, this study may contribute to a better understanding of cognitive and neuromuscular mechanisms involved in injury prevention and inform future preventive strategies in sports medicine.

## Background

Volleyball is a high-intensity sport involving repetitive movements such as jumping, rapid landings, and sudden directional changes, which place substantial mechanical stress on the knee joint and the anterior cruciate ligament (ACL) [1,2]. ACL injuries are among the most common and debilitating injuries in female volleyball players, significantly affecting athletic performance, quality of life, and long-term athletic prospects [3–5]. Research indicates that female volleyball players have a higher risk of non-contact ACL injuries compared to their male counterparts. These injuries most commonly occur during single-leg landings after spiking or during sudden defensive movements involving rapid directional changes. Biomechanical studies show that female athletes tend to land with reduced knee and trunk flexion and lower core muscle engagement than male athletes, which may contribute to increased anterior tibial shear force and greater stress on the ACL [1,2].

Abnormal landing patterns, including asymmetric knee joint angles, excessive femoral internal rotation, and increased dynamic knee valgus (DKV), represent high-risk movement profiles associated with ACL injuries [6,7]. Modifiable factors, such as weakness in the trunk stabilizers, quadriceps, and hamstrings, as well as muscle imbalances, further increase this risk [8,9]. Insufficient muscular support forces passive structures, such as ligaments and menisci, to absorb mechanical loads, significantly elevating the risk of injury [9]. To address this issue, screening tools such as the Tuck Jump Test have been developed to identify neuromuscular deficits, including DKV [9].

However, biomechanical and muscular factors alone may not fully explain the mechanisms underlying ACL injury risk. Because motor control relies on the coordinated function of the central nervous system (CNS), recent studies have increasingly emphasized the potential role of CNS function in ACL injury risk [10–12]. Neuroimaging studies using electroencephalography (EEG) and functional magnetic resonance imaging (fMRI) have reported alterations in the activation of brain regions involved in motor control, proprioception, and attention among athletes with characteristics associated with increased ACL injury risk [11,12]. These findings suggest that cognitive factors, such as decision-making, focus, and attentional control, may play an important role in injury prevention [11,12].

In real-game situations, jumping and landing occur under cognitively demanding and unpredictable conditions, requiring rapid decision-making, simultaneous processing of multiple stimuli, and swift motor responses [13]. This phenomenon, known as cognitive-motor interference or dual-task interference, occurs when cognitive demands compete with motor control for limited attentional resources. Cognitive challenges may impair the regulation of complex motor tasks, such as landing, and exacerbate movement deficiencies like DKV [13–16].

However, most existing injury prevention programs, such as FIFA 11+ and the PEP program, primarily focus on physical components, including muscle strengthening, balance training, and landing technique modification, while providing limited emphasis on cognitive domains such as attention, decision-making, and motor planning [17]. As a result, their applicability to cognitively demanding competitive environments may be limited. Thus, a training protocol that integrates neuromuscular performance and cognitive processing offers a potentially valuable approach to reducing ACL injury risk in female volleyball players, particularly those with DKV. Therefore, this study protocol addresses this gap by describing a randomized controlled trial designed to evaluate a neuromuscular-cognitive training program specifically developed for female volleyball athletes.

The primary objective of this trial will be to evaluate the effects of an eight-week neuromuscular-cognitive training protocol on single-leg landing mechanics, specifically peak knee valgus angle and frontal plane projection angle, during dual-task cognitive conditions in female volleyball players with DKV. The secondary objectives will be to assess the protocol’s effects on cortical brain activity patterns during motor tasks, measured by EEG, lower extremity muscle activation patterns during landing, assessed by surface electromyography (EMG), single-leg balance performance under both static and dynamic conditions, lower limb muscle strength (quadriceps, hamstrings, and hip abductors), and knee joint proprioception. Finally, this trial aims to contribute evidence regarding whether integrating cognitive challenges into neuromuscular training results in different outcomes compared with standard neuromuscular training alone, thereby informing future ACL injury prevention strategies in this high-risk athletic population.

## Methods

### Study design

This study protocol describes a two-arm, parallel-group randomized controlled trial (RCT) that will employ equal allocation (1:1) to evaluate the effects of an eight-week neuromuscular-cognitive training protocol on ACL injury risk factors in female volleyball athletes with DKV. Ethical approval was granted by the Research Ethics Committee of Bu-Ali Sina University (IR.BASU.REC.1402.076). This study has been registered with the Iranian Registry of Clinical Trials (IRCT20190224042827N5), registration date: 24 March 2024. The protocol integrates neuromuscular training principles [18], a cognitive intervention design model for ACL prevention [19], and the JUMP-ACL framework [20] to concurrently enhance motor and cognitive functions, with the aim of improving athletes’ adaptability in competitive environments where physical skills and rapid cognitive processing are critical. Assessments will be conducted at baseline (pre-intervention) and immediately following the eight-week intervention (post-intervention) to evaluate the protocol’s impact on primary and secondary outcomes.

### Setting

The study will be conducted among professional and collegiate female volleyball teams in Hamedan Province, Iran. All screening, training sessions, and outcome assessments will be performed in the Sports Rehabilitation Laboratory at Bu-Ali Sina University, equipped with 2D motion analysis systems, a foot scan plate, EEG and EMG equipment, and supervised by experienced sports medicine and rehabilitation professionals.

### Participants

#### Recruitment.

Female volleyball players aged 18–23 will be recruited from club-level and collegiate teams in Hamedan Province, Iran, through direct communication with coaches and team announcements. Information sheets outlining the study objectives, inclusion criteria, potential benefits, and the lead investigator’s contact details will be distributed to these teams. Interested players will be invited to the Sports Rehabilitation Laboratory at Bu-Ali Sina University for eligibility screening.

Screening will involve two cognitively loaded motor tests, the Tuck Jump Test and the Single-Leg Landing Task, to assess DKV. Only participants meeting the inclusion criteria based on screening results will provide informed consent and proceed to randomization. The research team will conduct recruitment and screening, with completion expected within one month.

### Maintenance strategy

Several strategies will be used to minimize participant dropout. A study coordinator will send reminder text messages before each scheduled training and assessment session. Training sessions will be embedded within each team’s existing practice schedule to minimize scheduling conflicts with competitions and academic commitments. Where feasible, makeup sessions will be offered for absences due to illness or minor scheduling conflicts, provided the participant does not meet the withdrawal threshold defined in the Exclusion Criteria. Reasons for dropout or withdrawal will be systematically recorded and reported using a CONSORT flow diagram. Participants who complete the full protocol will receive individualized feedback on their landing mechanics as a non-monetary incentive to continue engaging.

### Screening procedures

All potential participants will complete two screening assessments in the Sports Rehabilitation Laboratory:

*1- Tuck Jump Test with Cognitive Task:* Participants will perform continuous tuck jumps for 10 seconds while responding to visual stimuli. The test will be video-recorded and assessed for neuromuscular deficits associated with DKV [21].

*2- Single-Leg Landing Task with Cognitive Task:* Participants will perform single-leg landings from a 30-cm box while completing a visual discrimination task. Landing mechanics will be captured using 2D motion analysis techniques to quantify knee valgus angles. DKV will be defined as a peak knee valgus angle greater than 12 degrees during the single-leg landing task, consistent with established thresholds for increased ACL injury risk [22].

### Inclusion criteria

Participants must meet all of the following criteria:

- Female volleyball players aged 18–23 years
- Minimum of three years of competitive volleyball experience
- Currently training or competing at the club or collegiate level (minimum three training sessions per week)
- Presence of DKV confirmed by screening tests
- No history of ACL injury or reconstruction in either knee
- No lower extremity injury in the past six months requiring medical attention or time away from sport
- Able and willing to participate in training sessions three times per week for eight consecutive weeks
- Able to attend all assessment sessions

### Exclusion criteria

Participants will be excluded if they meet any of the following:

- History of ACL reconstruction or other knee ligament surgery
- Current lower extremity injury or pain that limits participation in volleyball activities
- History of concussion within the past six months
- Current participation in other structured injury prevention or cognitive training programs
- Unable or unwilling to provide informed consent
- Missing two consecutive or three non-consecutive scheduled training sessions.

### Informed consent

All eligible participants will receive detailed verbal and written information about the study objectives, procedures, potential risks and benefits, and their right to withdraw at any time without penalty. Written informed consent will be obtained from all participants before enrollment. All procedures involving participants in this study will follow the guidelines of the Research Ethics Committee of Bu-Ali Sina University (IR.BASU.REC.1402.076) and adhere to the 2013 Declaration of Helsinki.

### Qualifications of intervention providers

The intervention will be overseen by a PhD-level sports rehabilitation specialist with expertise in sports training, who will explain the study’s objectives to coaches and athletes, obtain informed consent, and supervise all assessments in the Sports Rehabilitation Laboratory at Bu-Ali Sina University. Day-to-day implementation of the training program will be conducted by trained team coaches under the supervision of the research team.

### Theoretical framework and design principles

This intervention protocol was developed based on the seven-step JUMP-ACL framework, incorporating evidence-based neuromuscular and cognitive training principles [18–20]. The intervention aims to address modifiable neuromuscular and biomechanical risk factors associated with ACL injury in young female volleyball athletes through a structured, progressive eight-week program. Previous literature has suggested that female volleyball athletes demonstrating DKV patterns may exhibit altered lower-extremity biomechanics associated with increased ACL loading during high-demand athletic tasks. Accordingly, the intervention is designed to improve movement mechanics and enhance lower-limb neuromuscular control under cognitively demanding conditions that approximate the attentional demands of real-game scenarios.

Based on previous literature regarding barriers to injury prevention program implementation in team sports and practical considerations identified through consultation with volleyball coaches, potential barriers to adherence include limited awareness of injury prevention strategies, time constraints within training schedules, and limited coach training in neuromuscular-cognitive intervention delivery. To address these barriers, the current protocol incorporates several implementation strategies, including comprehensive coach education, integration of training within existing team schedules, provision of detailed instructional materials, and emphasis on the cognitive training component to enhance athlete motivation and engagement.

Before intervention commencement, a two-hour standardized training workshop will be conducted for all participating team coaches and assistant coaches. The workshop will cover ACL injury mechanisms and prevention strategies, neuromuscular and cognitive contributors to injury risk in female athletes, demonstration and hands-on practice of intervention exercises, administration of cognitive tasks and integration of dual-task conditions, use of standardized performance evaluation checklists, identification of movement and cognitive errors, safety procedures, injury management and communication protocols, and strategies to maintain athlete motivation and adherence.

Following the workshop, coaches will be required to achieve a predefined competency threshold of 80% on a practical skills assessment, demonstrating adequate proficiency in exercise instruction, error identification, and progression decision-making.

### Intervention components

The training program combines two main components: neuromuscular training and cognitive training, both designed to address modifiable neuromuscular and biomechanical factors associated with ACL injury risk. Neuromuscular exercises aim to improve dynamic balance, increase strength of key lower limb muscles (especially hip abductors, quadriceps, and hamstrings), enhance proprioceptive function, and refine neuromuscular control during volleyball-specific high-risk movements such as jumping, landing, and cutting. Cognitive exercises are incorporated throughout neuromuscular training to create dual-task scenarios that challenge attention and decision-making under simultaneous cognitive and motor demands.

The cognitive training focuses on four critical areas essential for athletic performance. First, response selection and decision-making speed help athletes respond quickly to visual and auditory cues while performing movements, requiring rapid information processing and appropriate response selection. Second, working memory is trained by requiring athletes to recall movement sequences, task rules, or stimulus-response associations during exercises. Third, response inhibition involves practicing movement choices and inhibitions based on specific cues, requiring athletes to respond to target stimuli while suppressing irrelevant responses. Lastly, athletes train divided attention by monitoring environmental cues, such as volleyball or teammate movements, while maintaining postural control and executing dynamic exercises.

These cognitive exercises are designed to be simple, practical, progressive, and relevant to volleyball-specific demands. They are performed in pairs to facilitate peer feedback and encourage rapid and accurate responses.

All exercises in the program follow four core principles to ensure effectiveness and safety. The first is progressive overload, involving gradual increases in difficulty from simple to complex movements, single-task to dual-task conditions, and stable to less stable conditions when appropriate. The second is variety, as a wide range of exercises is included to maintain athlete engagement and target multiple performance aspects, such as strength, balance, power, and coordination. The third is repetition and continuity, ensuring sufficient practice volume and consistency to reinforce motor learning and potentially facilitate neural adaptations related to motor control. Lastly, expert supervision is essential, with trained staff consistently monitoring exercise execution to ensure proper technique, reduce inappropriate movement strategies, and provide immediate feedback and correction.

### Intervention protocol

The intervention will be conducted over eight consecutive weeks, with three supervised training sessions per week, each lasting 60 minutes (a total of 24 supervised sessions). These will be scheduled within the teams’ regular training times to minimize disruption and enhance adherence. Sessions will be performed on non-consecutive days to allow adequate recovery. Each session will follow a standardized structure: Warm-up (15 minutes), including dynamic stretching, light aerobic activity, and sport-specific movements according to established protocols; main training (30–40 minutes), focusing on progressive neuromuscular and cognitive exercises; and cool-down (10 minutes), consisting of static stretching of the primary lower-extremity muscles and breathing exercises to facilitate recovery.

The intervention was structured as a four-phase progressive training protocol, consisting of consecutive two-week stages with specific objectives and gradual increases in motor complexity and cognitive demands (Tables 1 and 2).

**Table 1:** Categorization of exercises based on movement plane, limb involvement, and equipment use.

*Exercise Name*	*Sagittal (S)*	*Frontal (F)*	*Transverse (T)*	*Double Leg*	*Single Leg*	*Equipment*
***Weeks 1–2: Controlled Movement Phase***						
Single-leg balance on a Bosu ball with induced valgus stress using a resistance band (knee flexed at 60°), Eyes open	*	*			*	Bosu ball, Resistance band, Mirror
Single-leg balance on a Bosu ball with induced valgus stress using a resistance band (knee flexed at 60°), Eyes closed	*	*			*	Bosu ball, Resistance band, Mirror
Wall jump	*			*		Mirror
Squat jump (60 degrees of knee flexion)	*			*		Flashcard, Mirror
Lunge jump	*			*		Mirror
Horizontal jump	*			*		Mirror
90-degree rotational jump			*	*		Mirror
Forward and backward line jump	*			*		Mirror
Barrier jumps	*			*		Barriers (10 cm), Mirror
Lateral and medial line jump		*		*		Mirror
Lateral jump with immediate vertical jump		*		*		Mirror
Forward drop landing with freeze	*			*		Step (30 cm), Flashcard, Mirror
***Weeks 3–4: Strategic Planning Phase***						
Single-leg stance on cushion, Eyes Open	*				*	Cushion balance, Cones
Single-leg balance on a Bosu ball with knee flexed at 60°, Eyes closed	*				*	Bosu ball
Triple horizontal jump and lateral jump	*			*		Step (20 cm)
180-degree rotational jump			*	*		–
Lateral and medial jumps over the barrier		*		*		Barrier (10 cm), Stroop cards
Forward and backward jumps over the barrier	*			*		Barrier (10 cm), Stroop cards
Jump over barriers with directional step landing	*	*		*		Barriers (10 cm), Steps (20 cm)
Forward drop jump and cones touch	*	*		*	*	Step (30 cm), Cones
Lateral drop jump and vertical jump		*		*		Step (30 cm)
Single-Leg vertical hop	*				*	Flashcards
Lateral hop hold		*			*	Flashcards
Lateral and medial single-leg hops on ladder		*			*	Ladder
***Weeks 5–6: Automatic Processing phase***						
Single-leg balance on a Bosu ball with knee flexed at 60° and trunk rotation, Eyes open	*		*		*	Bosu ball, Lightweight ball
Single-leg balance on a Bosu ball with knee flexed at 60°, Eyes closed	*				*	Bosu ball
Maximum horizontal and vertical jump	*			*		–
Tuck jump	*			*		Flashcards
Lunge jump with trunk rotation	*		*	*		–
90-degree lateral hops			*		*	–
Forward and backward hop over a line	*				*	Stroop cards
Lateral and medial hop over a line		*			*	Stroop cards
Multi-directional hop-hold	*	*			*	Flashcards
Lateral hop and barrier jump		*			*	Barriers (10 cm), Ball
Lateral drop landing with maximum vertical and horizontal jump	*	*		*		Step (30 cm)
Horizontal hop over barriers and step jump	*	*			*	Barrier (10 cm), Step (20 cm)
***Weeks 7–8: Transfer and Stabilization Phase***						
Single-leg balance on a Bosu ball with knee flexed at 60°, Eyes open	*				*	Bosu ball, Colored cards
Single-leg balance on a Bosu ball, Eyes open	*				*	Bosu ball, Ball
Stance on balance cushion, Open eyes	*				*	Cushion balance, Cones
Lunge jump on dual steps with trunk rotation	*		*	*		Steps (10 cm), Ball
Drop landing and Barrier jump, and Rotational step landing		*	*	*		Steps (30 cm), barrier (20 cm)
90°-degree medial hops			*		*	–
Crossover hop	*	*			*	Flashcards
Multi-directional hop-hop-hold	*	*			*	Flashcards
Lateral hop over barriers and step jump		*			*	Step (20 cm), Barriers (10 cm), Stroop cards
Medial hop over barriers and step jump		*			*	Step (20 cm), Barriers (10 cm), Stroop cards
Drop landing to maximum vertical jump	*				*	Step (30 cm), Flashcards
Drop landing to single-leg lateral jump with reversed cue		*			*	Step (30 cm)

**Table 2:** Description of physical exercises with cognitive tasks of study.

*Weeks 1–2: Controlled Movement Phase*				
**Goal:** Establish proper form of multi-plane movements and reduce dynamic valgus of the knee				
***Num***	***Exercise Name***	***Description***	***Cognitive Task Description***	***Repetition***
1	Single-leg balance on a Bosu ball with induced valgus stress using a resistance band (knee flexed at 60°), Eyes open	The athlete stands on one leg on a Bosu Ball with the stance knee flexed approximately 60 degrees. A partner applies an inward (valgus) force using a resistance band positioned around the athlete’s thigh. The athlete is instructed to maintain balance and resist the valgus force throughout the task.	*Week 1:* Internal focus on body alignment and joint stability *Week 2:* While the athlete maintains balance, the partner randomly calls out either a day of the week or a month of the year. The athlete must then verbally list the following (or preceding) days or months in correct order, depending on the partner’s instruction (forward or backward).	*Week 1:* 2 sets/ 20s *Week 2:* 2 sets/ 15s
2	Single-leg balance on a Bosu ball with induced valgus stress using a resistance band (knee flexed at 60°), Eyes closed	The athlete stands on one leg on a Bosu Ball with eyes closed and the stance knee flexed to approximately 60 degrees. A partner applies an inward (valgus) force using a resistance band positioned around the athlete’s thigh. The athlete is required to maintain postural stability and actively resist the applied valgus force throughout the task.	*Week 1:* Internal focus on body alignment and joint stability *Week 2:* Internal focus on body alignment and joint stability	*Week 1:* 2 sets/ 20s *Week 2:* 2 sets/ 20s
3	Wall jump	The athlete stands with feet hip-width apart and hands positioned beside the ears. The athlete initiates repeated vertical jumps, emphasizing smooth and controlled landings. Upon ground contact, the knees should flex forward naturally, and the movement should be performed rhythmically and without pause.	*Week 1:* Focus on motor control and technique using visual feedback from a mirror, partner, or coach. *Week 2:* Perform backward counting from randomly selected two-digit numbers, announced by the training partner.	*Week 1:* 2 sets/ 20s *Week 2:* 2 sets/ 20s
4	Squat jump (60 degrees of knee flexion)	The athlete begins in a hip-width stance and performs a vertical jump. During landing, the knees should remain aligned over the ankles and within the sagittal plane, avoiding any medial displacement or valgus collapse. Proper flexion of the knees _approximately 60 degrees_ and trunk should be maintained to absorb impact effectively. Arm swing is permitted to facilitate the jump, and the athlete should focus on achieving a soft, stable, and controlled landing.	*Week 1:* Focus on motor control and technique using visual feedback from a mirror, partner, or coach. *Week 2:* Identify and name a color, shape, animal, or object shown on a flashcard held by the partner.	*Week 1:* 2 sets/8 reps *Week 2:* 2 sets/6 reps
5	Lunge jump	The athlete assumes a hip-width stance and performs alternating lunge jumps. The legs should move along a straight forward-backward path, with the knees and thighs maintaining alignment and avoiding medial deviation.	*Week 1:* Focus on motor control and technique using visual feedback from a mirror, partner, or coach. *Week 2:* Solve simple arithmetic problems involving single-digit addition or multiplication.	*Week 1:* 2 sets/6 reps *Week 2:* 2 sets/5 reps
6	Horizontal jump	The athlete stands with feet shoulder-width apart and performs a forward jump. Emphasis is placed on proper landing mechanics, including balanced and controlled posture upon ground contact.	*Week 1:* Focus on motor control and technique using visual feedback from a mirror, partner, or coach. *Week 2:* React quickly to an auditory “go/no-go” signal provided by the partner.	*Week 1:* 2 sets/8 reps *Week 2:* 2 sets/6 reps
7	90-degree rotational jump	The athlete begins in a double-leg athletic stance and performs a vertical jump with a 90-degree rotation during the flight phase. The landing is executed with slight flexion at the hips and knees, using a toe-to-midfoot contact. The athlete should maintain the landing posture (double-leg stance) for at least 3 seconds to ensure stability.	*Week 1:* Focus on motor control and technique using visual feedback from a mirror, partner, or coach. *Week 2:* Respond oppositely to verbal commands indicating right or left rotation (i.e., reverse reaction).	*Week 1:* 2 sets/6 reps *Week 2:* 2 sets/6 reps
8	Forward and backward line jump	The athlete stands with feet shoulder-width apart and performs continuous jumps forward and backward over a marked line on the ground, maintaining balance and rhythm.	*Week 1:* Focus on motor control and technique using visual feedback from a mirror, partner, or coach. *Week 2:* Upon hearing a specific cue word (e.g., “up”) from the partner, the athlete quickly touches both shoulders.	*Week 1:* 2 sets/ 20s *Week 2:* 2 sets/ 10s
9	Barrier jumps	The athlete stands on both feet and performs forward jumps over three low obstacles (10 cm in height), spaced evenly along the ground. Proper landing and takeoff mechanics are emphasized throughout the sequence.	*Week 1:* Focus on motor control and technique using visual feedback from a mirror, partner, or coach. *Week 2:* Mentally add a series of three randomly selected single-digit numbers during or after the jump sequence.	*Week 1:* 2 sets/8 reps *Week 2:* 2 sets/6 reps
10	Lateral and medial line jump	The athlete stands with feet shoulder-width apart and performs rapid jumps side-to-side (laterally and medially) across a line marked on the floor. Emphasis is placed on symmetry and control.	*Week 1:* Focus on motor control and technique using visual feedback from a mirror, partner, or coach. *Week 2:* Name a person whose name starts with a specific letter given by the partner.	*Week 1:* 2 sets/ 20s *Week 2:* 2 sets/ 10s
11	Lateral jump with immediate vertical jump	The athlete performs a lateral jump from a central starting line. Upon landing, they immediately execute a vertical jump. This sequence challenges reactive strength and coordination.	*Week 1:* Focus on motor control and technique using visual feedback from a mirror, partner, or coach. *Week 2:* Answer a simple question (e.g., “What is the color of the sky?”) immediately after landing.	*Week 1:* 2 sets/8 reps *Week 2:* 2 sets/6 reps
12	Forward drop landing with freeze	The athlete steps off a raised step and lands on both feet, aiming to absorb the impact with deep knee flexion. The landing posture is held (frozen) for at least 3 seconds to promote postural control and joint stability.	*Week 1:* Focus on motor control and technique using visual feedback from a mirror, partner, or coach. *Week 2:* Land only when a predetermined visual or auditory cue (e.g., the number “3”) is presented.	*Week 1:* 2 sets/8 reps *Week 2:* 2 sets/8 reps
***Weeks 3–4: Strategic Planning Phase***				
**Goal:** Add a moderate cognitive load and enhance decision-making responsiveness				
***Num***	***Exercise Name***	***Description***	***Cognitive Task Description***	***Repetition***
1	Single-leg stance on cushion, Eyes Open	The athlete stands on one leg on a balance cushion and attempts to maintain postural stability.	In front of the athlete, five cones of different colors are placed. Upon verbal instruction from the partner (naming a color), the athlete bends down, touches the corresponding cone, and then returns to the initial upright position.	2 sets/ 20s
2	Single-leg balance on a Bosu ball with knee flexed at 60°, Eyes closed	The athlete stands on one leg on a Bosu ball with the knee flexed to approximately 60 degrees and eyes closed.	The partner applies gentle, unpredictable physical perturbations to the athlete’s body or the Bosu surface to challenge balance and proprioceptive control.	2 sets/ 20s
3	Triple horizontal jump and lateral jump	The athlete performs three consecutive forward (horizontal) jumps. After the third jump, they immediately perform a lateral jump onto either a right or left step.	The athlete performs a lateral jump based on verbal instruction provided by the partner.	2 sets/6 reps
4	180-degree rotational jump	Starting from a double-leg stance, the athlete performs a vertical jump while rotating the torso 180 degrees in mid-air. The athlete should land facing the opposite direction from the starting position.	Simultaneously, the athlete names a boy’s or girl’s name beginning with a randomly chosen letter of the alphabet, as prompted by the partner.	2 sets/6 reps
5	Lateral and medial jumps over the barrier	The athlete stands with feet shoulder-width apart and performs side-to-side jumps over a 10 cm barrier.	Performs a Stroop Color Naming Task; naming the color of the printed letters (not the written word).	2 sets/ 15s
6	Forward and backward jumps over the barrier	The athlete stands with feet shoulder-width apart and performs continuous forward and backward jumps over a 10 cm barrier.	Performs a Stroop Word Reading Task; reading the written word regardless of its color.	2 sets/ 15s
7	Jump over barriers with directional step landing	The athlete performs sequential jumps over three 10 cm barriers placed in a straight line. Upon completing the final jump, the athlete immediately jumps onto a step located in a randomly selected direction (right, left, or forward).	The movement is initiated by a verbal cue from the partner, who also announces the direction of the final jump. The athlete must accurately jump on the instructed step.	2 sets/5 reps
8	Forward drop jump and cones touch	Starting from an elevated step, the athlete performs a drop jump, immediately followed by a single-leg lateral jump in either direction. Upon jumping, the athlete bends down and touches the corresponding cone on that side with their hand.	Performs a reverse directional reaction: if the partner verbally cues “right,” the athlete must jump and respond to the left, and vice versa.	2 sets/8 reps
9	Lateral drop jump and vertical jump	The athlete begins by standing on a step and performs a lateral drop landing to either the left or right side. Immediately after landing, the athlete executes a vertical jump.	Performs a random visual reaction task: the partner stands in front of the athlete and indicates a direction using hand gestures (e.g., points to the right), and the athlete must react in the opposite direction (i.e., jump left).	2 sets/8 reps
10	Single-Leg vertical hop	The athlete balances on one leg and performs a vertical hop. Upon landing, the goal is to maintain postural stability and correct lower limb alignment.	The partner presents flashcards containing different colors or objects. The athlete must verbally say the color or object shown on each card during or immediately after the hop.	2 sets/8 reps
11	Lateral hop hold	Starting from a single-leg athletic stance, the athlete performs a lateral hop and lands using a toe-to-midfoot contact with slight knee flexion. The landing position is held isometrically for at least 3 seconds to develop balance and joint stability.	The partner presents flashcards displaying three single-digit numbers. The athlete is instructed to memorize the numbers during the task and recite them in the correct order after completing the movement.	2 sets/6 reps
12	Lateral and medial single-leg hops on a ladder	The athlete stands on one leg in the second square of an agility ladder. They hop laterally to the adjacent square and then return medially by hopping back two squares.	Respond to simple verbal questions, such as “What color is the sky?”.	2 sets/5 reps
***Weeks 5–6: Automatic Processing phase***				
**Goal:** Automation of movement form under cognitive pressure				
***Num***	***Exercise Name***	***Description***	***Cognitive Task Description***	***Repetition***
1	Single-leg balance on a Bosu ball with knee flexed at 60° and trunk rotation, Eyes open	The athlete stands on one leg on a Bosu ball with the knee flexed to approximately 60 degrees. While maintaining balance, the athlete performs repeated trunk rotations to the left and right while holding a lightweight ball in both hands.	During the task, the athlete simultaneously answers simple arithmetic problems involving addition, subtraction, or multiplication, as verbally prompted by the partner.	2 sets/ 15s
2	Single-leg balance on a Bosu ball with knee flexed at 60°, Eyes closed	The athlete stands on one leg on a Bosu ball with eyes closed and the stance knee flexed at approximately 60 degrees. The partner applies mild, random perturbations to the Bosu ball to challenge the athlete’s postural control and proprioception.	While maintaining balance, the athlete counts backwards by twos, starting from a random two-digit number provided by the partner.	2 sets/ 20s
3	Maximum horizontal and vertical jump	The athlete performs a maximal effort horizontal (forward) jump, immediately followed by a maximal vertical jump upon landing. The movement is executed in a fluid, continuous manner.	The jump sequence is initiated in response to a go/no-go visual stimulus, provided by the partner. The athlete must only begin the movement upon recognizing the “go” signal.	2 sets/8 reps
4	Tuck jump	The athlete stands with feet shoulder-width apart and performs repeated tuck jumps by explosively jumping upward and simultaneously bringing the knees toward the chest during flight. Emphasis is placed on maintaining symmetry and soft landings.	Five single-digit numbers are sequentially shown to the athlete during the task. The athlete is instructed to compute and verbally announce the total sum of the numbers at the end of the jumping sequence.	2 sets/ 10s
5	Lunge jump with trunk rotation	The athlete begins in a hip-width stance and performs alternating lunge jumps. During each jump, the trunk rotates actively in the direction of the front leg. Proper knee alignment and trunk control are emphasized throughout the motion.	The athlete performs a backward count, subtracting by threes from a randomly provided three-digit number verbally by the partner.	2 sets/ 15s
6	90-degree lateral hops	From a single-leg athletic stance, the athlete performs a lateral hop, lands with slight knee flexion using a toe-to-midfoot strategy, and then immediately hops medially to return to the starting point. The sequence is repeated for a predetermined duration or repetition count.	The athlete responds to a simple verbal question (e.g., “What color is the sky?”).	2 sets/6 reps
7	Forward and backward hop over a line	The athlete stands on one leg and performs consecutive forward and backward hops over a line marked on the floor. Landing mechanics and balance are closely monitored.	A Stroop task is performed concurrently, in which the athlete is presented with color-word stimuli and must respond based on the meaning of the word (e.g., ignore the color of the text and say the word itself).	2 sets/ 15s
8	Lateral and medial hop over a line	The athlete stands on one foot and performs lateral (side-to-side) hops over a designated line. The focus is on speed, symmetry, and postural control.	The athlete performs a Stroop task (color reading) by stating the color of the ink used to print a conflicting word, thereby engaging executive function and response inhibition.	2 sets/ 15s
9	Multi-directional hop-hold	From a single-leg stance, the athlete hops in one of four directions (forward, backward, left, right) as indicated, lands with control, and returns to the starting point.	A task-switching test is used: the athlete is shown a series of cards containing words with matching or mismatching font colors. Based on cues, they must either read the word or say the color, requiring continuous cognitive shifting.	2 sets/ 10s
10	Lateral hop and barrier jump	The athlete stands on one leg, positioned between two 10 cm barriers. They hop laterally toward one barrier and perform an additional hop to clear it, then return to the center.	The partner stands in front and throws a ball to either the left or the right. The athlete initiates movement after recognizing the ball’s direction and must hop in the same direction as the throw.	2 sets/6 reps
11	Lateral drop landing with maximum vertical and horizontal jump	The athlete begins on an elevated step and performs a lateral drop landing onto both feet. Immediately after landing, a maximal vertical jump is executed, followed by a maximal forward jump in sequence.	The movement is initiated in response to an auditory cue (e.g., whistle), requiring a quick sensory-motor reaction.	2 sets/6 reps
12	Horizontal hop over barriers and step jump	The athlete performs three single-leg forward hops over a series of 10 cm barriers. At the end of the sequence, they must jump onto a 20 cm step located either in front, to the left, or the right.	Both the initiation of movement and the step direction are determined by a partner’s verbal command, requiring auditory processing and directional decision-making during dynamic motion.	2 sets/5 reps
***Weeks 7–8: Transfer and Stabilization Phase***				
**Goal:** Transferring learning to real-game situations and increasing neural flexibility				
***Num***	***Exercise Name***	***Description***	***Cognitive Task Description***	***Repetition***
1	Single-leg balance on a Bosu ball with knee flexed at 60°, Eyes open	The athlete balances on one leg atop a Bosu ball, with their knee flexed at approximately 60 degrees and eyes open, focused on a wall.	While positioned in front of a wall displaying multiple-colored targets, the athlete maintains balance on a single leg atop a Bosu ball. In response to verbal cues provided by a partner (e.g., “yellow”), the athlete dynamically reaches with the upper limb to touch the corresponding-colored circle. This task is designed to enhance visual discrimination, decision-making, and upper-limb coordination under postural control demands.	1 set/ 20s
2	Single-leg balance on a Bosu ball, Eyes open	While standing on one leg with knees slightly bent on a Bosu ball, the athlete maintains balance and catches a ball passed by the partner, then returns it.	During the ball-passing task, the athlete answers simple verbal questions (e.g., “What day is it today?”), engaging attention and verbal processing alongside physical stability.	1 set/ 15s
3	Stance on balance cushion, Open eyes	The athlete stands on one leg on a soft balance cushion with eyes open. Four colored cones are placed anterior, posterior, and lateral to the athlete.	Upon verbal command from the partner indicating a color, the athlete touches the corresponding cone using the free foot, integrating balance control with working memory and response execution.	1 set/ 20s
4	Lunge jump on dual steps with trunk rotation	The athlete starts between two 10 cm-high steps and performs a plyometric lunge with an explosive vertical jump, landing with each foot on a separate step while rotating the trunk.	Upon landing, the athlete receives a ball passed by the partner, requiring anticipatory timing, visuomotor coordination, and dual-task balance.	2 sets/4 reps
5	Drop landing and Barrier jump, and Rotational step landing	The athlete drops from a step to the floor, immediately jumps over a 20 cm hurdle, and lands on a secondary step while rotating the trunk 90°, resulting in a directional change of gaze.	While performing the movement, the partner verbally presents three random single-digit numbers. The athlete must calculate and announce their sum upon completion of the movement.	2 sets/5 reps
6	90°-degree medial hops	The athlete performs a single-leg hop while rotating 90° medially mid-air, landing with controlled knee flexion on the same leg. The posture is held for a minimum of 3 seconds. Direction is opposite to the stance leg (e.g., right leg initiates leftward rotation).	During execution, the athlete responds to simple verbal questions (e.g., “What color is the sky?”), maintaining cognitive engagement.	2 sets/6 reps
7	Crossover hop	From a single-leg stance, the athlete performs three consecutive crossover hops over a central line (e.g., right leg: left → right → left), maintaining balance in a deep flexion hold for at least 3 seconds on the final landing.	The partner presents three or five single-digit numbers during the movement, which the athlete must retain and recall in correct sequence after completing the task.	2 sets/6 reps
8	Multi-directional hop-hop-hold	The athlete performs two consecutive hops in a designated direction (forward, backward, left, or right) on one leg and holds the final landing position for 3 seconds.	During the task, the athlete responds to simultaneous visual cues indicating a color and a number, integrating visual processing and cognitive flexibility.	2 sets/ 10s
9	Lateral hop over barriers and step jump	The athlete stands laterally and hops over three 10 cm barriers, concluding with a vertical jump onto the elevated step.	Performs a Stroop color-naming task by stating the ink color of printed words, requiring inhibition of automatic reading.	2 sets/8 reps
10	Medial hop over barriers and step jump	The athlete stands medially and hops over three 10 cm barriers, concluding with a vertical jump onto the elevated step.	Engages in a Stroop word-reading task, reading the word itself regardless of its ink color, stimulating cognitive inhibition and selective attention.	2 sets/8 reps
11	Drop landing to maximum vertical jump	The athlete drops from a step, lands on one foot, and immediately performs a maximal vertical jump with the same leg.	The partner reads out a sequence of three single-digit numbers during the movement; the athlete recalls and states the numbers in order after completion.	2 sets/6 reps
12	Drop landing to single-leg lateral jump with reversed cue	The athlete drops from a step with one leg, then immediately performs a lateral jump to the side opposite of the instructed landing leg.	The athlete receives a landing cue from the partner (e.g., “Land on right”), but must perform the movement using the opposite foot, introducing a cognitive inhibition and reverse-reaction component.	2 sets/8 reps

In the first phase (Weeks 1–2), referred to as the Controlled Movement Phase, the primary goal was to establish proper multi-plane movement patterns and improve movement strategies associated with dynamic knee valgus through foundational exercises performed under high levels of external feedback. Training emphasized sagittal-plane movements with controlled frontal-plane components, simple single-task neuromuscular exercises accompanied by real-time verbal and visual feedback, and the introduction of basic cognitive tasks. Exercises were performed in pairs to allow peer observation and feedback, mainly on stable surfaces with bilateral support when needed, and at low to moderate intensity.

The second phase (Weeks 3–4), referred to as the Strategic Planning Phase, aimed to introduce moderate cognitive demands by emphasizing decision-making and motor planning during movement. This stage incorporated a greater proportion of single-leg exercises, integration of dual-task conditions combining motor and cognitive demands, and more complex cognitive challenges such as choice reaction time and stimulus discrimination. External feedback was reduced to encourage reliance on internal sensory feedback mechanisms, while unstable surfaces and dynamic balance challenges were introduced. Exercises were performed at moderate intensity and complexity.

In the third phase (Weeks 5–6), called the Automatic Processing Phase, the objective was to develop more efficient motor control under cognitive load and improve dual-task proficiency. Athletes received minimal external feedback and were encouraged to self-monitor and correct their performance. Training involved complex multi-directional movements across multiple planes, high cognitive load tasks requiring divided attention, and increased speed and intensity. Sport-specific movement patterns resembling volleyball actions were emphasized, with a focus on efficient and fluid movement execution.

Finally, the fourth phase (Weeks 7–8), designated as the Transfer and Stabilization Phase, sought to facilitate the transfer of acquired skills to competition-like situations, emphasizing frontal-plane single-leg control and reinforcement of learned motor adaptations. This stage involved maximum cognitive load with unpredictable, game-like stimulus conditions, frontal-plane dominant single-leg tasks mimicking volleyball-specific movements such as lateral cutting and defensive slides, and integration of volleyball equipment and scenarios. Exercises were performed at high intensity and speed, under fatigue and time pressure, to facilitate application of learned skills during dynamic sport situations.

Athletes will progress to the next training phase only when they consistently meet predefined progression criteria across three consecutive sessions. Movement quality will be assessed by a trained coach using a standardized checklist, ensuring appropriate lower-extremity alignment according to predefined movement-quality criteria. Cognitive performance will also be evaluated, requiring athletes to commit no more than three predefined cognitive errors per session during tasks involving stimulus responses or rule-based challenges. Athletes must maintain movement quality under dual-task conditions without clinically meaningful deterioration compared with single-task performance and execute tasks at the prescribed speed and intensity while meeting cognitive demands. Since progression is individualized, athletes who do not meet the criteria after completing the planned two-week phase will remain at the same level with modified or alternative exercises of similar complexity until the requirements are achieved, thereby supporting adequate motor learning, confidence development, and minimizing inappropriate progression-related injury risk. All progression decisions will be documented using standardized forms and reviewed weekly by the supervising sports rehabilitation specialist to ensure safety.

Training intensity will be indirectly quantified using the session rating of perceived exertion (session-RPE) method [23]. Approximately 30 minutes after each session, each participant will rate her perceived exertion using the modified Borg CR-10 scale; session-RPE will then be multiplied by session duration (minutes) to calculate session training load. Weekly training load will be reviewed by the supervising sports rehabilitation specialist alongside the movement-quality and cognitive-error checklists to identify unusual increases in perceived exertion and inform individualized progression decisions.

### Control group protocol

The control group will receive an active control intervention consisting of the same neuromuscular training exercises as the experimental group but performed exclusively under single-task conditions without cognitive challenges. This design will control for exercise volume, intensity, trainer attention, and social interaction, thereby estimating the additional contribution of cognitive training integration. Training will be delivered three times per week for eight weeks, with each session lasting approximately 60 minutes and following an identical structure to the experimental group: a 15-minute warm-up, 30–40 minutes of main training, and a 10-minute cool-down. Progression will follow the same four-phase structure, with gradual increases in exercise difficulty, intensity, and complexity. Instructional methods will include verbal cueing, demonstration, and corrective feedback; all will be provided with a single-task focus to ensure consistency across groups while eliminating additional cognitive demands.

Both groups will continue their regular volleyball team training sessions, competitions, and usual physical activities throughout the 8-week intervention period. All participants will be instructed to maintain consistent training and competition schedules and report any changes in activity level, injuries, or other relevant health factors.

To enhance motivation and adherence to the eight-week neuromuscular-cognitive training program, each female volleyball player will set an individualized goal based on her personal objectives. These goals will be used to enhance engagement without altering the standardized exercise prescription. A simple checklist will be provided to each participant to track progress, monitor ongoing performance, and record the frequency and type of cognitive and neuromuscular errors, encouraging motivation through ongoing personalized feedback. To avoid mental fatigue and maintain engagement, the protocol includes varied neuromuscular and cognitive training components. Team coaches will actively participate in delivering the training, guiding sessions, and ensuring high-quality execution.

During the intervention period, participants will be instructed to avoid performance-enhancing stimulants, tobacco, non-steroidal anti-inflammatory drugs (NSAIDs), or other analgesics within 24 hours before each training session to minimize potential effects on pain perception and physical performance. They will be permitted to continue their regular team training sessions and maintain their usual physical activity and dietary habits throughout the study.

### Timeline

**Fig 1:**
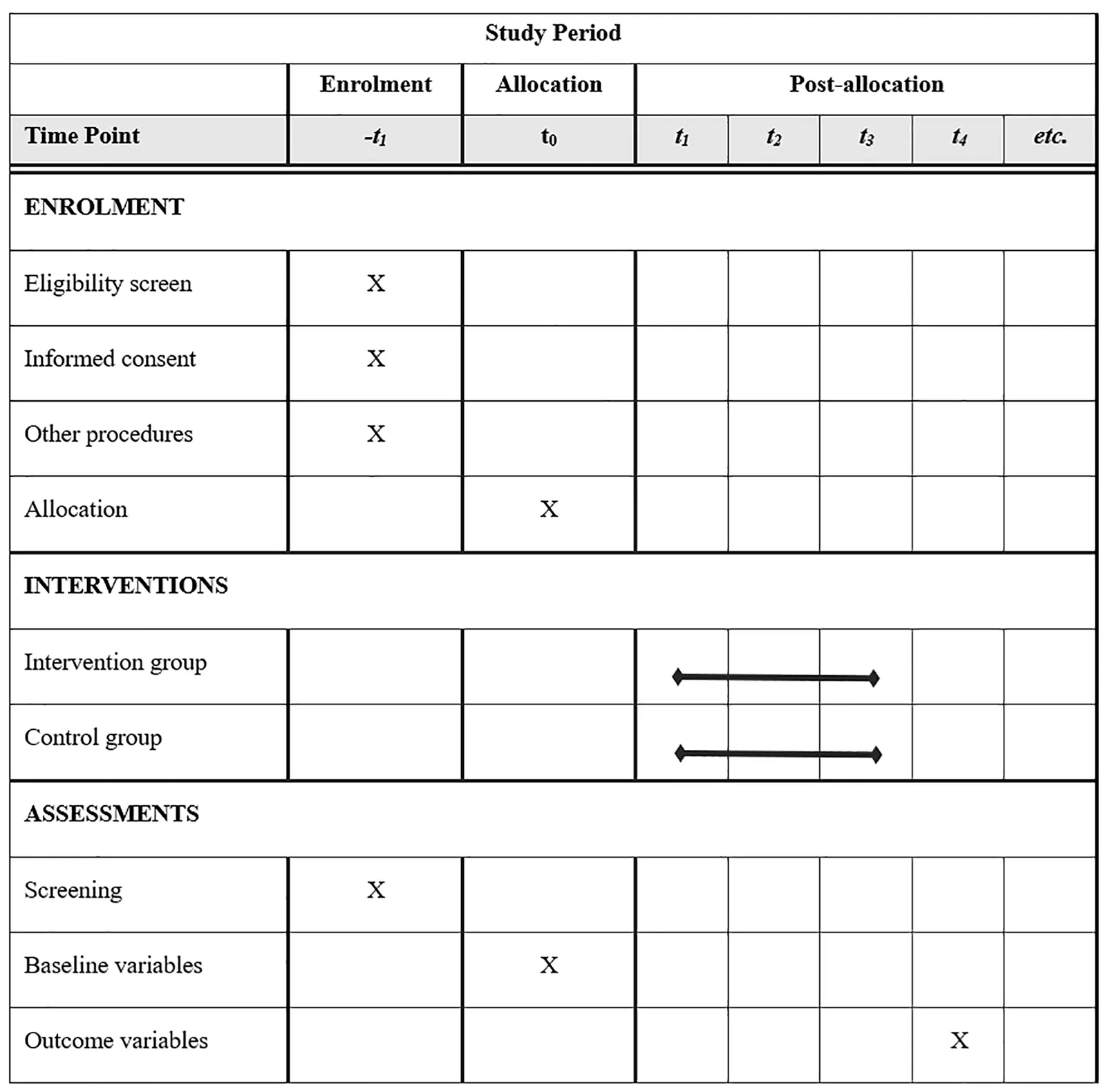
Schedule of enrolment, interventions, and assessments. − t_1_, enrolment and screening week; t_0_, allocation and baseline assessment; t_1_ - t_3_, interventions; t_4_, post-interventions assessment.

The study timeline and schematic overview, including participant enrolment procedures, baseline and post-intervention outcome assessments, and the eight-week intervention period, are presented in Fig 1.

### Outcomes

Participants will first receive detailed information about the study procedures, ethical considerations, and potential risks before providing informed consent and completing a personal information form. They will then undergo standardized instructions and a familiarization session, followed by a warm-up to standardize physical readiness and minimize injury risk. Assessments will be conducted 72 hours before the start of the eight-week neuromuscular-cognitive training protocol (pre-test) and within 72 hours after the final session (post-test) to evaluate its effects on modifiable ACL injury-related risk factors in female volleyball players with DKV. All assessments will take place in the Sports Rehabilitation Laboratory at Bu-Ali Sina University under standardized temporal and environmental conditions, with precise measures to control external variables and ensure accurate evaluation.

The primary outcome is the change in two-dimensional landing kinematics, particularly movement patterns associated with DKV, during single-leg landing under two conditions: with and without a concurrent cognitive task. Secondary outcomes include electrocortical brain activity measured by electroencephalography (EEG), lower limb muscle activity assessed by surface electromyography (sEMG), single-leg balance, lower limb muscle strength, and knee joint proprioception. These measures will determine the intervention’s impact on neuromuscular, cognitive, and biomechanical factors associated with ACL injury risk.

### Harms

All self-reported adverse events, unintended intervention effects, or changes in participants’ health status, including pain, discomfort, musculoskeletal symptoms, or injuries, will be documented and reported to the principal investigator for review and appropriate action.

### Sample size

The sample size was calculated using G*Power software (version 3.1) for a repeated-measures ANOVA (within-between interaction) to detect changes in DKV angle at initial contact. The effect size was derived from the group × time interaction reported for DKV at initial contact in a previous neuromuscular training study with dual cognitive tasks [24] (partial η² = 0.19), converted to an effect size f = 0.48. Based on a significance level (α) of 0.05, statistical power (1-β) of 0.95, and an assumed correlation of r = 0.5 between repeated measures, a minimum total of 18 participants was required. The required sample size was initially determined based on the primary outcome measure (the DKV angle) to ensure sufficient statistical power. To adjust for multiple secondary outcome assessments and an estimated 20% attrition rate, we inflated the target enrollment to 30 participants, who will be randomly allocated in a 1:1 ratio to the intervention and control groups.

### Assignment of interventions: Allocation

#### Sequence generation.

Participants will be randomized using a computer-generated random number sequence. They will be allocated to either the intervention or control group in a 1:1 ratio. To ensure group balance and minimize assignment predictability, block randomization with variable block sizes (for example, 4 and 6) will be employed. An independent researcher, not involved in recruitment, data collection, or intervention delivery, will generate the randomization sequence before the study begins.

### Concealment mechanism

To conceal allocation and prevent selection bias, the randomization sequence will be sealed in opaque, tamper-proof, sequentially numbered envelopes. Each envelope will contain a card indicating group assignment corresponding to the participant’s number. The envelopes will be opened only after completion of baseline assessments. An independent individual, not involved in study implementation, will prepare and manage the envelopes.

### Implementation

An independent investigator, not involved in participant recruitment, assessment, or intervention administration, will generate the randomization sequence and maintain exclusive access to the randomization list. After confirming eligibility and obtaining informed consent, this investigator will randomize participants. The study coordinator, blinded to group assignment, will enroll eligible female volleyball athletes with DKV and schedule assessments. Due to the nature of the intervention, trainers cannot be blinded to group allocation. The allocation process is designed to minimize selection and performance bias throughout the study.

### Assignment of interventions: Blinding

This study will be conducted as a participant- and assessor-blinded randomized controlled trial. Participants will be informed that they are receiving training, but will not know whether the program includes neuromuscular training with or without cognitive components. Given the structural similarity between protocols, participants may be unlikely to determine their group assignment. To minimize communication between groups, intervention and control sessions will be scheduled on separate training days. Outcome assessors will remain blinded to group assignments. Participants will be instructed not to disclose intervention-related information during evaluations.

### Data collection methods

To evaluate landing kinematics, electrocortical brain activity, and muscle activity, a single-leg landing test will be administered under two experimental conditions: with and without a concurrent cognitive task. After reflective markers and EMG sensors are placed on the participant’s preferred kicking leg (determined using a ball-kicking test) and the EEG cap is fitted, participants will perform the landing test three times with cognitive load and three times without cognitive load. During the test, frontal-plane knee valgus and sagittal-plane knee flexion angles, as well as trunk flexion and lateral flexion angles, will be captured using two cameras positioned for anterior and lateral views. Muscle activity in the vastus medialis, vastus lateralis, biceps femoris, semitendinosus, and gluteus medius will be measured using surface EMG. Electrocortical brain activity will be recorded via 19 EEG channels at theta and alpha-2 frequency bands [25].

To begin the test, participants will cross their arms over their chest to minimize upper-limb movement while standing on a 30 cm-high step [22]. The heel of the dominant foot will be positioned at the edge of the step to limit preparatory movement. Participants will look forward to a wall-mounted screen. After the presentation of the final arrow cue, the participant will perform a single-leg landing on either the right or left target area marked on the floor, based on the direction of the final arrow. The test will also be conducted without cognitive load as a control condition. If the participant cannot maintain balance or demonstrates loss of control (for example, requires additional support, steps outside the target area, or loses postural stability), the trial will be considered invalid and excluded from analysis [26].

After completing the landing tests, participants will stand on a foot scan platform to perform a 30-second balance assessment under eyes-open and eyes-closed conditions. Subsequently, knee muscle strength (flexors and extensors) and hip muscle strength (abductors and adductors) will be measured using a hand-held dynamometer [17]. Knee joint proprioception at 60° of flexion (non-weight-bearing) will be assessed using a goniometer.

In addition, health-related quality of life will be evaluated before and after the intervention using the SF-12 questionnaire [27]. The analysis criterion for all variables will be the change from baseline values.

### Data management

Two trained sports rehabilitation specialists will collect and enter all data electronically using secure, password-protected software. Each participant will receive a unique identification number to ensure anonymity. The principal investigator (PI), in collaboration with the data management and analysis team, will perform data quality control, checking for missing values, out-of-range entries, and duplicate records. This team will operate separately from the intervention delivery team to minimize potential bias and ensure data integrity, adherence to pre-specified statistical procedures, and reproducibility.

### Statistical methods

Descriptive statistics, including means and standard deviations, will summarize the data. The Shapiro-Wilk test will assess data normality. Baseline characteristics will be summarized descriptively between groups. Repeated-measures ANOVA will be used to evaluate the group × time interaction effect as the primary indicator of intervention effectiveness (training vs. control; pre-test vs. post-test). Pairwise comparisons following significant interactions will be conducted with Bonferroni correction to control type I error. The five secondary outcome domains (cortical brain activity, lower-limb muscle activation, single-leg balance, muscle strength, and knee joint proprioception) will be interpreted as exploratory outcomes and hypothesis-generating findings. Partial eta squared will quantify effect sizes, interpreted as small (0.01), medium (0.06), or large (0.14). All analyses will be performed using SPSS version 27 with α = 0.05 and a 95% confidence level.

Given the modest sample size, supplementary analyses will be conducted. Baseline values will be reviewed descriptively, and if clinically meaningful baseline imbalance is observed, ANCOVA models adjusting for baseline values will be performed as sensitivity analyses. Missing data patterns will be assessed, and linear mixed models with appropriate covariance structures will be considered as sensitivity analyses to evaluate robustness under missing-at-random assumptions. Any meaningful discrepancy between supplementary analyses and the primary ANOVA will be reported and discussed.

To assess clinical significance and intervention effectiveness, minimal clinically important difference (MCID) and minimal detectable change (MDC) will be calculated at a 95% confidence level. The MDC will be derived using the standard error of measurement (SEM), as defined by the following formulas [28]:


$$\begin{array}{c} \text{SEM }=\;{\text{SD}}_{\text{pre}}\;\times \;\sqrt{(\text{1}-\text{ ICC})\;} \end{array}$$

$$\begin{array}{c} {\text{MDC}}_{\%\text{9}\text{5}}\;=\;\text{1}.\text{96 }\times \sqrt{\text{2}\;\times \text{ SEM}} \end{array}$$

$$\begin{array}{c} {\text{MCID}}_{\text{RCI}}=\;\text{1}.\text{96 }\times \;{\text{SD}}_{\text{pre}}\;[\sqrt{\left(\text{2}\times (\text{1}-\text{ICC})\right)}] \end{array}$$

The reliable change index (RCI) will be calculated to determine whether observed changes exceed measurement error. In these equations, SDpre denotes the standard deviation of pre-test scores in the training group, and ICC denotes the intraclass correlation coefficient (test-retest reliability). MCID_RCI_ will be used to determine the minimum clinically significant change. Reliability coefficients from previously reported studies in comparable populations will be used.

## Discussion

This study aims to investigate the effects of a neuromuscular-cognitive training protocol on ACL injury-related risk factors and athletic performance in female volleyball players with DKV. By comparing the neuromuscular-cognitive training protocol with neuromuscular training alone, the study will evaluate the additional contribution of cognitive components to injury prevention strategies.

The intervention integrates motor and cognitive training under dynamic, sport-specific conditions, based on current evidence regarding neuromuscular and cognitive factors associated with injury risk. Unlike many previous injury-prevention approaches that primarily targeted isolated physical parameters, such as strength or balance, this protocol adopts a holistic strategy to enhance motor control, sensorimotor function, and cognitive performance simultaneously. This approach aims to replicate the complex and unpredictable demands of competitive volleyball, where ACL injury risk may increase, thereby addressing both biomechanical and cognitive-related risk factors.

This study investigates the integration of cognitive elements within neuromuscular training, reflecting the complex demands of competitive volleyball and potentially informing future ACL injury prevention strategies. The protocol targets female volleyball players with DKV, a high-risk yet understudied group. The randomized controlled trial design strengthens internal validity, and the intervention’s feasibility may support future implementation in sports and clinical settings. Blinded outcome assessors will be used to minimize observer bias. The study addresses the need for effective prevention programs in women’s sports amid increased participation and competitive demands.

As a study protocol, the intervention’s effectiveness remains to be determined after completion of outcome assessments. The focus on female volleyball players with DKV may limit generalizability to other populations or sports. The lack of objective biomechanical load-monitoring tools (such as GPS and accelerometry) constrains precise training intensity quantification; this limitation is partially addressed through session-RPE monitoring and standardized progression checklists, although these subjective and semi-quantitative measures cannot fully replace objective external load measures. Additionally, menstrual cycle-related variations and other physiological factors were not controlled, which may influence neuromuscular performance and intervention response in female athletes.

### Dissemination plans

Results will be disseminated through peer-reviewed journals, including open-access platforms when possible, to ensure broad accessibility for researchers, clinicians, and relevant stakeholders. A plain-language summary, accompanied by visual materials outlining key findings and practical implications, will be shared with study participants and their coaches to support the translation of research findings into sports practice.

## Supporting information

S1 File Protocol for ethics committee-ENG version. (PDF)

S2 File Protocol for ethics committee-Persian version. (PDF)

S3 File SPIRIT 2025 editable checklist. (DOCX)
