# Supplementary material for: Effects of neuromuscular-cognitive training on ACL injury risk factors in female volleyball athletes with dynamic knee valgus: Protocol for a randomized controlled trial
Source: PLoS One. 2026 Aug 25;21(8):e0353944. doi: 10.1371/journal.pone.0353944 (PMC13505893; doi:10.1371/journal.pone.0353944)
Supplement: Supplementary file 1 [file pone.0353944.s001.pdf]

Study Protocol

**Title**	Comparison of the Effects of Neuromuscular Training with and Without Cognitive Interventions on Landing Mechanics, Proprioception, Balance, Strength, and Electrocortical Activity in Female Athletes at Risk of ACL Injury
**Student Information**	PhD candidate, Department of Exercise Rehabilitation, Faculty of Sports Science, Bu-Ali Sina University, Hamedan, Iran. E-mail: f.ramezani@phe.basu.ac.ir
**Supervisors**	Farzaneh Saki, Associate Professor, Department of Exercise Rehabilitation, Faculty of Sports Science, Bu-Ali Sina University, Hamedan, Iran. E-mail: f_saki@basu.ac.ir<br>Mohammadreza Zoghipaydar, Associate Professor, Department of Psychology, Faculty of Economic and Social Science, Bu-Ali Sina University, Hamedan, Iran. E-mail: m.r.zoghipaidar@basu.ac.ir
**Study Location**	Bu-Ali Sina University, Hamedan, Iran
**Funding**	No funding was received for this study.

Problem Definition and Rationale for the Study

The knee joint, positioned centrally within the kinetic chain of the lower limb, is subjected to substantial mechanical loads during athletic activities [1]. Anterior cruciate ligament (ACL) injury is among the most common lower-extremity injuries in athletes; annually, approximately 200,000 ACL injuries occur in the United States alone [2], and nearly 90% of affected individuals ultimately undergo ACL reconstruction (ACLR) [3]. This injury is particularly prevalent among participants in team sports such as soccer, futsal, handball, volleyball, and basketball. ACL injury can lead to numerous complications, including short-term consequences such as pain, stiffness, and joint effusion, and long-term consequences such as joint instability, osteoarthritis, meniscal damage, and functional impairments [4], all of which necessitate prolonged and complex treatment. Moreover, athletes are forced to withdraw from training and competition, which may impose additional psychological burdens. Both surgical and rehabilitation-based treatments impose substantial financial costs on individuals and society. Beyond the high annual treatment expenses, ACL injury also results in loss of sports participation and, in many cases, loss of an entire competitive season [5, 6].

Jumping and landing are the most common mechanisms of non-contact ACL injury [7], with more than one-third of non-contact ACL injuries occurring immediately after landing from a jump [7]. Landing is a complex motor task influenced by biomechanical, neuromuscular, and neurocognitive deficits. A stiff landing pattern characterized by minimal flexion of the trunk, hip, knee, and ankle increases peak ground reaction forces and elevates ACL injury risk [8]. Poor trunk control and excessive femoral motion can increase internal rotation and adduction of the femur, ultimately resulting in dynamic knee valgus (DKV) and increased ACL loading [9]. Limited ankle dorsiflexion during landing is also associated with reduced sagittal-plane knee flexion and

increased frontal-plane DKV, further elevating strain on the ACL [10]. Neuromuscular deficits involving the trunk, lower leg, quadriceps, and ligament-dominant movement patterns are considered modifiable risk factors strongly associated with ACL injury [11]. Ligament dominance or DKV occurs when the motor control strategies adopted by the athlete fail to provide adequate dynamic knee stability, causing the athlete to rely on passive ligamentous structures rather than muscular support to absorb ground reaction forces, thereby increasing injury risk [12]. The tuck jump test, which includes repeated plyometric activities, is commonly used to identify neuromuscular risk factors associated with ACL injury [11].

ACL injuries frequently occur during scenarios requiring complex sensorimotor coordination, highlighting the critical role of the central nervous system (CNS) in maintaining functional joint stability [13]. A study comparing electrocortical activity between athletes at high and low risk for ACL injury reported distinct resting-state electrocortical patterns in high-risk athletes. Specifically, high-risk athletes demonstrated reduced continuous electrocortical activity in frontal theta and central/parietal alpha-2 frequency bands, suggesting suboptimal neural function and impaired information transfer between functional networks responsible for attentional control and sensorimotor processing [14]. In a prospective fMRI study, female soccer players who later sustained an ACL injury exhibited reduced resting-state functional connectivity between the left primary somatosensory cortex (responsible for processing sensory input) and the right posterior cerebellum (involved in motor control) compared with their uninjured teammates [15].

Proprioception is defined as neuromuscular coordination and the ability to integrate sensory information to maintain awareness of joint and body position [16]. Given the essential role of proprioception in achieving joint stability [17], participation in neuromuscular training programs may improve proprioception, muscular strength, and biomechanical performance during jump-landing tasks, thereby reducing ACL injury risk [16].

In team sports, athletes must process information rapidly due to time and space constraints [18]. Responding to unpredictable stimuli and attentional limitations affects athletic performance and may increase injury risk [18]. In sports where ACL injuries are common, athletes do not pre-plan their movements; instead, they react to sudden external perturbations [19]. During competition, athletes must execute motor patterns while simultaneously attending to opponents' movements, ball position, and auditory cues from teammates and coaches. These cognitive demands and divided attention may increase ACL injury risk [20, 21]. A study examining the effects of divided attention on lower-limb kinematic variability in female athletes found that under dual-task conditions, athletes demonstrated reduced knee flexion and increased DKV [22]. Despite the clear association between cognitive challenges and injury occurrence, most injury-prevention programs focus solely on motor performance and neglect cognitive factors [23].

Dual-task cognitive challenges, based on the limited-capacity theory of attention, involve performing two or more cognitive and motor tasks simultaneously [22]. Each task requires a portion of attentional resources, and interference occurs when the combined attentional demands exceed the individual's processing capacity or when tasks rely on shared neural resources [24]. Training that incorporates dual-task cognitive challenges may help overcome CNS processing limitations and facilitate the consolidation of correct motor patterns within cortical networks [25]. Despite the availability of numerous injury-prevention programs, ACL injury rates remain

substantial. Integrating neurocognitive approaches may help address existing gaps in current prevention strategies. Effective injury-prevention programs should challenge the CNS and enhance the integration of visual and proprioceptive information to maximize transferability to real-game conditions [20]. Simultaneous neuromuscular and cognitive-dual-task training may reduce attentional limitations and promote automaticity of motor patterns during competition. To develop valid and effective training protocols for injury prevention, cognitive task demands must be considered, as they may influence the effectiveness of ACL injury-prevention programs.

According to the researcher's review, few studies have incorporated cognitive dual-task training to improve landing mechanics, proprioception, balance, strength, and brain functional connectivity in athletes at high risk for ACL injury. The findings of the present study may contribute to the development of training protocols, identification of injury risk factors, and enhancement of the effectiveness of ACL injury-prevention programs. Therefore, this study aims to examine the effects of eight weeks of neuromuscular training with and without cognitive dual-task challenges on landing mechanics, proprioception, balance, strength, electrocortical activity, and electromyography in female volleyball athletes with dynamic knee valgus.

Research Objectives

General Objective

To compare the effectiveness of eight weeks of neuromuscular training with and without cognitive interventions on landing mechanics, proprioception, balance, strength, electrocortical activity, and electromyography in female athletes at risk of ACL injury.

Specific Objectives

- To compare the effects of neuromuscular training with and without cognitive interventions on landing mechanics in female athletes at risk of ACL injury.
- To compare the effects of neuromuscular training with and without cognitive interventions on knee proprioception in female athletes at risk of ACL injury.
- To compare the effects of neuromuscular training with and without cognitive interventions on balance in female athletes at risk of ACL injury.
- To compare the effects of neuromuscular training with and without cognitive interventions on lower-limb strength in female athletes at risk of ACL injury.
- To compare the effects of neuromuscular training with and without cognitive interventions on electrocortical activity in female athletes at risk of ACL injury.
- To compare the effects of neuromuscular training with and without cognitive interventions on electromyographic activity in female athletes at risk of ACL injury.

Research Hypotheses

- Eight weeks of neuromuscular training with and without cognitive interventions will affect landing mechanics in female athletes at risk of ACL injury.
- Eight weeks of neuromuscular training with and without cognitive interventions will affect knee proprioception in female athletes at risk of ACL injury.
- Eight weeks of neuromuscular training with and without cognitive interventions will affect balance in female athletes at risk of ACL injury.
- Eight weeks of neuromuscular training with and without cognitive interventions will affect lower-limb strength in female athletes at risk of ACL injury.
- Eight weeks of neuromuscular training with and without cognitive interventions will affect electrocortical activity in female athletes at risk of ACL injury.
- Eight weeks of neuromuscular training with and without cognitive interventions will affect electromyographic activity in female athletes at risk of ACL injury.

Methods

Study Design

A randomized controlled trial (RCT).

Inclusion Criteria

Participants were eligible for inclusion if they met all of the following criteria:

- Female volleyball players aged 18-23 years.
- A minimum of three years of competitive athletic experience.
- Presence of dynamic knee valgus (DKV) confirmed through screening tests, including:
- Observable DKV during the tuck jump test with a cognitive task.
- Dynamic knee valgus angle greater than 12° during a single-leg landing task with a cognitive challenge.
- No lower limb injury in the past six months requiring medical treatment or cessation of sports activity.
- No history of knee surgery or central nervous system disorders.
- Ability and willingness to participate in training sessions three times per week for eight consecutive weeks.
- Availability to attend pre-test and post-test assessment sessions.

Exclusion Criteria

- Participants were excluded if they met any of the following conditions:
- Presence of lower limb pain or injury that prevented participation in sports activities.
- Absence from two consecutive or three non-consecutive training sessions during the intervention period.
- Experiencing pain or sustaining an injury during the training protocol.

Sampling Method and Sample Size

The target population consisted of female volleyball players from club-level and university-level teams in Hamedan. Participants were recruited by identifying active teams and coordinating with coaches and team managers.

Sample size was calculated using G*Power version 3.1 with a Repeated Measures ANOVA (within-between interaction). Considering an alpha level of 0.05, statistical power of 0.85, and an effect size of 0.31 (based on DKV angle data from Hamoongard et al., 2022), the minimum required sample size was estimated at 26 participants. Accounting for an anticipated 15% dropout rate, the sample size was increased to 30 participants. Ultimately, 15 female athletes with DKV were allocated to each of the training and control groups.

Study Procedure

To ensure voluntary participation, written informed consent was obtained from all participants. All potential risks were explained, and participants were informed that they were free to withdraw at any time without penalty or loss of access to routine care or services. Participation was free of charge, and all collected data were kept confidential.

After enrollment, participants were assigned to either the experimental or control group. The experimental group received the neuromuscular cognitive training program for eight weeks, while the control group received neuromuscular training only. Randomization was performed using a Random Number Generator. Allocation concealment was ensured using sequentially numbered, opaque, sealed envelopes.

Intervention

Corrective Intervention

The intervention consisted of two main components: neuromuscular training and cognitive training.

Neuromuscular Training

Designed to improve dynamic balance, strengthen key lower limb muscles (particularly hip abductors, quadriceps, and hamstrings), enhance proprioception, and correct high-risk movement patterns. Exercises included multiplanar movements, balance tasks, and jump landing drills under varying conditions.

Cognitive Training

Integrated into motor tasks to create dual task conditions requiring simultaneous cognitive processing. Four cognitive domains were targeted:

Rapid decision making (responding to visual and auditory stimuli).

Working memory (recalling and executing motor or numerical sequences).

Response inhibition (ignoring irrelevant stimuli).

Divided attention (simultaneously tracking the ball or teammates while performing movements).

Cognitive tasks were designed to be simple, progressive, sport specific, and performed in pairs to enhance peer feedback.

Training Structure

The intervention consisted of 24 sessions over eight weeks (three sessions per week). Each 60-minute session was held on non-consecutive days and integrated into regular team practice schedules to enhance adherence.

Session Components

Warm up (15 min): Dynamic stretching, light aerobic activity, volleyball-specific drills.

Main training (30-40 min): Progressive neuromuscular and cognitive tasks.

Cool down (10 min): Static stretching and breathing exercises.

Training Phases

The protocol included four progressive two-week phases:

Phase 1: Movement Control (Weeks 1-2)

Focus on correcting sagittal plane movement patterns and reducing DKV.

Simple, double-leg tasks with high visual and verbal feedback on stable surfaces.

Phase 2: Strategic Planning (Weeks 3-4)

Increased cognitive load.

Introduction of single-leg tasks, decision making, and reaction to stimuli.

Reduced external feedback to promote proprioceptive reliance.

Phase 3: Automatic Processing (Weeks 5-6)

Goal: automatization of motor patterns under dual task conditions.

Multidirectional, single-leg, high-speed, and high-intensity tasks with substantial cognitive load.

Phase 4: Transfer and Consolidation (Weeks 7-8)

Goal: transfer of skills to real game scenarios.

Single-leg frontal plane movements, unpredictable stimuli, and volleyball-specific scenarios (e.g., defensive slides, rapid direction changes).

High-intensity tasks performed under fatigue and time pressure.

Progression Criteria

Progression to the next phase required meeting predefined performance criteria for three consecutive sessions:

Motor domain: Knee alignment and movement quality assessed by a trained coach.

Cognitive domain: Error rate, movement quality under dual task conditions, response speed, and accuracy.

Participants who did not meet the criteria remained in the same phase and performed alternative exercises of similar difficulty. All decisions were documented in standardized forms and reviewed weekly by a sports specialist.

Control Group Intervention

The control group participated in an active intervention consisting of the same neuromuscular exercises as the experimental group but performed exclusively under single-task conditions without cognitive components. Session structure, frequency, duration, and intensity were identical across groups to control for training volume, coach attention, and social interaction.

Control group training also followed the same four progressive phases, but without cognitive load. Instructional strategies included verbal cues, demonstrations, and corrective feedback, focusing solely on physical execution. This design allowed isolation of the cognitive component's effect.

Statistical Analysis

Statistical analyses will be performed using SPSS version 27. Descriptive statistics (mean ± SD) will be reported for continuous variables. The Shapiro-Wilk test will assess normality. Independent t-tests will compare baseline demographic variables between groups.

A Repeated Measures ANOVA will examine group (training vs. control) × time (pre-test vs. post-test) interactions. Bonferroni-adjusted pairwise comparisons will control for Type I error. Effect sizes will be calculated using Partial Eta Squared.

Clinical significance will be evaluated using the Minimal Clinically Important Difference (MCID) and Minimal Detectable Change (MDC) at a 95% confidence level. MDC will be calculated using the Standard Error of Measurement (SEM):

$$\mathrm{SEM} = \mathrm{SD}_{\mathrm{pre}} \times \sqrt{1 - \mathrm{ICC}}$$

$$\mathrm{MDC}_{95} = 1.96 \times \sqrt{2} \times \mathrm{SEM}$$

MCID will be calculated using the Reliable Change Index (RCI):

$$\mathrm{MCID}_{\mathrm{RCI}} = 1.96 \times \mathrm{SD}_{\mathrm{pre}} \times \sqrt{2(1 - \mathrm{ICC})}$$

Where:

- $\mathrm{SD}_{\mathrm{pre}}$= standard deviation of pre-test values in the training group
- ICC= intraclass correlation coefficient (test-retest reliability)

References

.١ Cowling EJ, Steele JR. Is lower limb muscle synchrony during landing affected by gender? Implications for variations in ACL injury rates. Journal of Electromyography and Kinesiology. 2001;11(4):263-268.

.٢ Levangie PK, Norkin CC. Joint structure and function: a comprehensive analysis: FA Davis 2011.

.٣ Smith TO, Davies L, Hing CB. Early versus delayed surgery for anterior cruciate ligament reconstruction: a systematic review and meta-analysis. Knee surgery, sports traumatology, arthroscopy. 2010;18:304.٣١١-

.٤ Hewett TE, Ford KR, Hoogenboom BJ, et al. Understanding and preventing acl injuries: current biomechanical and epidemiologic considerations-update 2010. North American journal of sports physical therapy: NAJSPT. 2010;5(4):234.

.٥ Imwalle LE, Myer GD, Ford KR, et al. Relationship between hip and knee kinematics in athletic women during cutting maneuvers: a possible link to noncontact anterior cruciate ligament injury and prevention. Journal of strength and conditioning research/National Strength & Conditioning Association. 2009;23(8):2223.

.٦ Quatman CE, Hewett TE. The anterior cruciate ligament injury controversy: is "valgus collapse" a sex-specific mechanism? British journal of sports medicine. 2009;43(5):328-335.
.٧ Giesche F, Wilke J, Engeroff T, et al. Are biomechanical stability deficits during unplanned single-leg landings related to specific markers of cognitive function? Journal of science and medicine in sport. 2020;23(1):82-88.
.٨ DiStefano LJ, Root HJ, Frank BS, et al. Implementation strategies for ACL injury prevention programs. ACL injuries in the female athlete: causes, impacts, and conditioning programs. 2018:625-639.
.٩ Alentorn-Geli E, Myer GD, Silvers HJ, et al. Prevention of non-contact anterior cruciate ligament injuries in soccer players. Part 1: Mechanisms of injury and underlying risk factors. Knee surgery, sports traumatology, arthroscopy. 2009;17:705-729.
.١٠ Hart JM, Grindstaff TL, Beazell JR, et al. Assessment of ankle dorsiflexion range of motion restriction. Athletic Training & Sports Health Care. 2009;1(1):7-8.
.١١ Herrington L, Myer GD, Munro A. Intra and inter-tester reliability of the tuck jump assessment. Physical Therapy in Sport. 2013;14(3):152-155.
.١٢ Hewett TE, Myer GD, Ford KR, et al. Biomechanical measures of neuromuscular control and valgus loading of the knee predict anterior cruciate ligament injury risk in female athletes: a prospective study. The American journal of sports medicine. 2005;33(4):492-501.
.١٣ Grooms DR, Onate JA. Neuroscience application to noncontact anterior cruciate ligament injury prevention. Sports Health. 2016;8(2):149-152.
.١٤ Bonnette S, Diekfuss JA, Grooms DR, et al. Electrocortical dynamics differentiate athletes exhibiting low- and high-ACL injury risk biomechanics. Psychophysiology. 2020;57(4):e13530.
.١٥ Diekfuss JA, Grooms DR, Yuan W, et al. Does brain functional connectivity contribute to musculoskeletal injury? A preliminary prospective analysis of a neural biomarker of ACL injury risk. Journal of science and medicine in sport. 2019;22(2):169-174.
.١٦ Mandelbaum BR, Silvers HJ, Watanabe DS, et al. Effectiveness of a neuromuscular and proprioceptive training program in preventing anterior cruciate ligament injuries in female athletes: 2-year follow-up. The American journal of sports medicine. 2005;33(7):1003-1010.
.١٧ Nagai T, Schilaty ND, Strauss JD, et al. Analysis of lower extremity proprioception for anterior cruciate ligament injury prevention: current opinion. Sports Medicine. 2018;48(6):1303-1309.
.١٨ Liu R, Liu J, Ma X, et al. Effect of FIFA 11+ intervention on change of direction performance in soccer and futsal players: A systematic review and meta-analysis. International Journal of Sports Science & Coaching. 2021;16(3):862-872.
.١٩ Junge A, Dvorak J. Injury risk of playing football in Futsal World Cups. British journal of sports medicine. 2010;44(15):1089-1092.
.٢٠ Dai B, Cook RF, Meyer EA, et al. The effect of a secondary cognitive task on landing mechanics and jump performance. Sports biomechanics. 2018;17(2):1.٢٠٥-٩٢

.۲۱ Nazary-Moghadam S, Salavati M, Esteki A, et al. Reliability of kinematic measures in subjects with anterior cruciate ligament deficiency during dual-task walking. Journal of bodywork and movement therapies. 2017;21(4):852-859.
.۲۲ Almonroeder TG, Kernozek T, Cobb S, et al. Divided attention during cutting influences lower extremity mechanics in female athletes. Sports biomechanics. 2019;18(3):264-276.
.۲۳ Monfort SM, Pradarelli JJ, Grooms DR, et al. Visual-spatial memory deficits are related to increased knee valgus angle during a sport-specific sidestep cut. The American journal of sports medicine. 2019;47(6):1488-1495.
.۲٤ Pashler H. Dual-task interference in simple tasks: data and theory. Psychological bulletin. 1994;116(2):220.
.۲٥ Ruthruff E, Van Selst M, Johnston JC, et al. How does practice reduce dual-task interference: Integration, automatization, or just stage-shortening? Psychological research. 2006;70:125-142.
