# Supplementary material for: Effects of neuromuscular-cognitive training on ACL injury risk factors in female volleyball athletes with dynamic knee valgus: Protocol for a randomized controlled trial
Source: PLoS One. 2026 Aug 25;21(8):e0353944. doi: 10.1371/journal.pone.0353944 (PMC13505893; doi:10.1371/journal.pone.0353944)
Supplement: Supplementary file 2 [file pone.0353944.s002.pdf]

پروتکل مطالعاتی

**عنوان**	مقایسه تأثیر تمرینات عصبی-عضلانی با و بدون مداخلات شناختی بر مکانیک فرود، حس عمقی، تعادل، قدرت و فعالیت الکتروکورتیکال در ورزشکاران زن مستعد آسیب ACL
**مشخصات دانشجو**	فرزانه رمضانی، دانشجوی دکتری، گروه توانبخشی ورزشی، دانشکده علوم ورزشی، دانشگاه بوعلی سینا، همدان، ایران.
**مشخصات اساتید**	فرزانه ساکی، گروه توانبخشی ورزشی، دانشکده علوم ورزشی، دانشگاه بوعلی سینا، همدان، ایران.<br>محمدرضا ذوقی پایدار، گروه روانشناسی، دانشکده علوم اقتصادی و اجتماعی، دانشگاه بوعلی سینا، همدان، ایران.
**مکان**	دانشگاه بوعلی سینا همدان
**نهاد مالی**	ندارد.

تعریف مسئله و بیان ضرورت انجام پژوهش

مفصل زانو به علّت قرارگیری در نقطه میانی زنجیره حرکتی اندام تحتانی، ازجمله مفاصلی است که طی فعّالیّت‌های ورزشی تحت‌فشارهای زیادی قرار می‌گیرد.[۱] آسیب رباط صلیبی قدامی (ACL) یکی از شایع‌ترین آسیب‌های اندام تحتانی در ورزشکاران بشمار می‌آید؛ به‌طوری‌که طبق آمار سالیانه ۲۰۰ هزار آسیب ACL به‌تنهایی در ایالات متحده آمریکا اتفاق می‌افتد[۲] و حدود ۹۰ درصد از بیماران، متقاضی بازسازی رباط صلیبی قدامی (ACLR) می‌باشند.[۳] این آسیب در بین افرادی که در ورزش‌های تیمی مانند فوتبال، فوتسال، هندبال، والیبال و بسکتبال شرکت می‌کنند شایع است. آسیب ACL می‌تواند عوارض متعددی را بر جای بگذارد؛ از جمله عوارض کوتاه‌مدت آن می‌توان به درد، سفتی، تورم مفصلی و از جمله عوارض بلندمدت آن می‌توان به بی‌ثباتی مفصل، استئوآرتریت، آسیب‌های منیسک و اختلالات عملکردی اشاره نمود[۴] و به همین دلیل درمان پیچیده و طولانی‌مدتی نیاز دارد. همچنین ورزشکار آسیب‌دیده را از میادین مسابقه و تمرین دور نگه می‌دارد که این امر خود می‌تواند عوارض روحی متفاوتی بر آنان داشته باشد. از طرف دیگر درمان این آسیب خواه به‌صورت جراحی و خواه به‌صورت توان‌بخشی هزینه سنگینی را بر دوش فرد و جامعه می‌گذارد. آسیب ACL علاوه بر هزینه درمان سالیانه زیاد، موجب ازدست‌دادن مشارکت ورزشی و حتی ازدست‌دادن فصل ورزشی می‌شود.[۵،۶]

پرش و فرود شایع‌ترین مکانیسم آسیب غیرتماسی ACL است.[۷] بیش از یک‌سوم آسیب‌های غیرتماسی ACL اغلب پس از فرود از پرش رخ می‌دهد.[۷] تکنیک فرود پیچیده است و تحت‌تأثیر نقایص بیومکانیکی، عصبی-عضلانی و عصبی شناختی است، بنابراین فرود سفت با حداقل خمیدگی در تنه، لگن، زانو و مچ پا باعث اوج نیروی واکنش زمین و افزایش خطر آسیب ACL می‌شود.[۸] ناتوانی در کنترل تنه و حرکات ران می‌تواند منجر به افزایش چرخش داخلی و اداکشن استخوان ران شود که در نهایت منجر به والگوس پویا زانو (DKV) و افزایش نیروهای وارد بر ACL می‌شود.[۹] محدودیت دامنه حرکتی دورسی فلکشن مچ پا هنگام فرود نیز با کاهش خم‌شدن زانو در صفحه ساژیتال و افزایش DKV در صفحه فرونتال همراه است که کشش بیش از حد بر ACL وارد می‌کند.[۱۰] نقایص عصبی-عضلانی (تنه، ساق پا، چهار سر ران و غلبه رباط) به‌عنوان عوامل خطر قابل‌اصلاح در نظر گرفته می‌شوند که به‌شدت با آسیب ACL مرتبط هستند.[۱۱] نقص غلبه رباط زانو یا DKV

زمانی رخ می‌دهد که استراتژی‌های کنترل حرکتی اتخاذ شده توسط ورزشکار، ثبات داینامیکی کافی برای مفصل زانو ایجاد نکند. در این شرایط ورزشکار از رباط‌های زانو به‌جای عضلات، جهت جذب نیروی عکس‌العمل زمین و نیروهای تماسی استفاده می‌کند و خطر وقوع آسیب افزایش می‌یابد.[۱۲] تست پرش تاک شامل فعالیت‌های پلایومتریک مکرر است که عوامل عصبی-عضلانی مرتبط با آسیب ACL را شناسایی می‌کند.[۱۱]

آسیب ACL اغلب در طول سناریوهایی رخ می‌دهد که نیاز به هماهنگی حسی-حرکتی پیچیده دارند که نمونه‌ای از نقش حیاتی سیستم عصبی مرکزی (CNS) در حفظ ثبات عملکردی مفصل است.[۱۳] نتایج یک مطالعه که فعالیت الکتروکورتیکال ورزشکاران با ریسک بالا و پایین آسیب ACL را مقایسه کرده بود، نشان داد که ورزشکاران با ریسک بالا آسیب ACL، نسبت به ورزشکاران با ریسک پایین آسیب ACL، الگوی متفاوتی از فعالیت الکتروکورتیکال در حالت استراحت داشته‌اند. به طور دقیق‌تر، ورزشکاران با ریسک بالا آسیب، فعالیت الکتروکورتیکال کمتر پیوسته‌ای را در باندهای فرکانسی تتا فرونتال و آلفا-۲ سنترال و پریتال نشان داده‌اند. این تفاوت در الگوهای الکتروکورتیکال ممکن است نشان‌دهنده عملکرد عصبی نامناسب باشد، و انتقال اطلاعات عصبی بین شبکه‌های عملکردی مختلف مربوط به کنترل توجه و پردازش حسی-حرکتی را برای ورزشکاران پرخطر سخت‌تر می‌کند.[۱۴] در یک مطالعه آینده‌نگر با استفاده از تصویربرداری رزونانس مغناطیسی عملکردی (fMRI)، زنانی که در فصل مسابقات فوتبال به آسیب ACL مبتلا شده بودند با هم‌تیمی‌های سالم خود مقایسه شدند.[۱۵] تصویربرداری رزونانس مغناطیسی عملکردی کاهش اتصال عملکردی در حالت استراحت را بین قشر سوماتوسنسوری اولیه سمت چپ (ناحیه‌ای که اطلاعات حسی عصبی را پردازش می‌کند) و لوب خلفی مخچه در سمت راست (ناحیه‌ای که با کنترل حرکتی مرتبط است) قبل از آسیب ACL نشان داد. حس عمقی به‌عنوان هماهنگی عصبی-عضلانی و توانایی یکپارچه کردن اطلاعات حسی در جهت حفظ آگاهی وضعیت مفاصل و بخش‌های بدن تعریف می‌شود.[۱۶] به دلیل نقش سیستم حس عمقی در دسترسی به ثبات مفصلی[۱۷] ممکن است شرکت در برنامه تمرینات عصبی-عضلانی باعث تغییر در حس عمقی، قدرت ورزشکار و عملکرد بیومکانیکی تکنیک پرش و فرود شود که این مهم می‌تواند منجر به کاهش خطر آسیب ACL شود.[۱۶]

به دلیل محدودیت‌های زمانی و مکانی در ورزش‌های تیمی، ورزشکاران بایستی در سریع‌ترین زمان ممکن فرایندهای پردازش اطلاعات را انجام دهند.[۱۸] واکنش به محرک‌های پیش‌بینی‌نشده و محدودیت‌های توجه در ورزش‌های تیمی بر عملکرد ورزشکاران تأثیر می‌گذارد و می‌تواند فرد را در معرض آسیب قرار دهد.[۱۸] در ورزش‌های تیمی که آسیب ACL شایع است، ورزشکاران جهت حرکت از قبل تصمیم‌گیری نمی‌کنند، بلکه در پاسخ به اغتشاشات شدید خارجی در حال حرکت هستند.[۱۹] یک ورزشکار در شرایط مسابقه باید الگوهای حرکتی را هم‌زمان باتوجه‌به حرکات حریف، موقعیت توپ، صدای هم‌تیمی‌ها و مربی انجام دهد که این شرایط به دلیل تقسیم توجه و چالش‌های شناختی تحمیل شده بر ورزشکار می‌تواند فرد را در معرض آسیب ACL قرار دهد.[۲۰,۲۱] پژوهشی با هدف تأثیر تقسیم توجه بر تغییرپذیری کینماتیک اندام تحتانی در زنان ورزشکار انجام شد، در شرایط تقسیم توجه ورزشکاران فلکشن کمتر و افزایش DKV را نشان دادند.[۲۲] با وجود ارتباط آشکار بین چالش‌های شناختی و وقوع آسیب، مورد قابل‌توجه این است که در تمرینات پیشگیری از آسیب تنها عملکرد حرکتی فرد مورد ارزیابی قرار گرفته است و به فاکتورهای شناختی اهمیتی داده نمی‌شود.[۲۳]

تکالیف شناختی دوگانه طبق نظریه ظرفیت محدود توجه به‌عنوان انجام هم‌زمان دو یا چند وظیفه شناختی و حرکتی تعریف می‌شود.[۲۲] در واقع انجام هر تکلیف نیاز به استفاده بخشی از ظرفیت توجه دارد و درصورتی‌که منبع پردازش اطلاعات در مغز یکی باشد و یا نیازهای توجه تکالیف از ظرفیت کلی پردازش اطلاعات فرد بیشتر باشد، تداخل در تکالیف رخ خواهد داد.[۲۴] تمرینات همراه با تکالیف شناختی دوگانه باعث غلبه بر

محدودیت‌های پردازش اطلاعات در CNS و نهادینه‌شدن الگوی حرکتی صحیح در قشر خاکستری مغز می‌شود.[۲۵] با وجود برنامه‌های پیشگیری از آسیب متنوع، میزان وقوع آسیب‌های ACL هنوز هم قابل‌توجه است، احتمالاً استفاده از رویکردهای عصبی شناختی بتواند شکاف موجود در برنامه‌های پیشگیری از آسیب را پر نماید. تمرینات پیشگیری از آسیب جهت حداکثر تعمیم‌پذیری با شرایط مسابقه بایستی CNS را به چالش بکشند و در یکپارچگی اطلاعات بینایی و حس عمقی نقش داشته باشند.[۲۰] احتمالاً انجام هم‌زمان تمرینات عصبی-عضلانی با تکالیف شناختی باعث کاهش محدودیت‌های توجه و خودکار شدن الگوهای حرکتی ورزشکاران در شرایط مسابقه می‌شود. اگر هدف توسعه اجرای پروتکل‌های تمرینی معتبر در جهت پیشگیری از آسیب باشد، نیازهای تکالیف شناختی بایستی در نظر گرفته شوند که ممکن است محدودیت تأثیرگذاری برنامه‌های تمرینی پیشگیری از آسیب ACL را مشخص کند. بر طبق جستجوی محقق، کمتر مطالعه‌ای از تمرینات تکالیف شناختی جهت بهبود مکانیک فرود ورزشکاران با ریسک بالا آسیب ACL، حس عمقی، تعادل، قدرت و اتصال عملکردی مغز استفاده کرده است. نتایج حاصل از این پژوهش موجب توسعه پروتکل‌های تمرینی، شناسایی عوامل خطر آسیب و بهبود اثرگذاری برنامه‌های تمرینی پیشگیری از آسیب خواهد شد؛ لذا هدف از پژوهش حاضر بررسی تأثیر هشت هفته تمرینات عصبی-عضلانی با و بدون تکالیف دوگانه شناختی بر مکانیک فرود، حس عمقی، تعادل، قدرت و فعالیت الکتروکورتیکال و الکترومایوگرافی در ورزشکاران والیبالیست زن با نقص ولگوس پویای زانو می‌باشد.

اهداف پژوهش

هدف کلی: مقایسه اثربخشی هشت هفته تمرینات عصبی-عضلانی با و بدون مداخلات شناختی بر مکانیک فرود، حس عمقی، تعادل، قدرت، فعالیت الکتروکورتیکال و الکترومایوگرافی ورزشکاران زن مستعد آسیب ACL

اهداف اختصاصی

مقایسه تأثیر تمرینات عصبی-عضلانی با و بدون مداخلات شناختی بر مکانیک فرود ورزشکاران زن مستعد آسیب ACL

مقایسه تأثیر تمرینات عصبی-عضلانی با و بدون مداخلات شناختی بر حس عمقی زانو ورزشکاران زن مستعد آسیب ACL

مقایسه تأثیر تمرینات عصبی-عضلانی با و بدون مداخلات شناختی بر تعادل ورزشکاران زن مستعد آسیب ACL

مقایسه تأثیر تمرینات عصبی-عضلانی با و بدون مداخلات شناختی بر قدرت اندام تحتانی ورزشکاران زن مستعد آسیب ACL

مقایسه تأثیر تمرینات عصبی-عضلانی با و بدون مداخلات شناختی بر فعالیت الکتروکورتیکال ورزشکاران زن مستعد آسیب ACL

مقایسه تأثیر تمرینات عصبی-عضلانی با و بدون مداخلات شناختی بر فعالیت الکترومایوگرافی ورزشکاران زن مستعد آسیب ACL

فرضیه‌های پژوهش

هشت هفته تمرینات عصبی-عضلانی با و بدون مداخلات شناختی بر مکانیک فرود ورزشکاران زن مستعد آسیب ACL تأثیر دارد.

هشت هفته تمرینات عصبی-عضلانی با و بدون مداخلات شناختی بر حس عمقی زانو ورزشکاران زن مستعد آسیب ACL تأثیر دارد.

هشت هفته تمرینات عصبی-عضلانی با و بدون مداخلات شناختی بر تعادل ورزشکاران زن مستعد آسیب ACL تأثیر دارد.

هشت هفته تمرینات عصبی-عضلانی با و بدون مداخلات شناختی بر قدرت اندام تحتانی ورزشکاران زن مستعد آسیب ACL تأثیر دارد.

هشت هفته تمرینات عصبی-عضلانی با و بدون مداخلات شناختی بر فعالیت الکتروکورتیکال ورزشکاران زن مستعد آسیب ACL تأثیر دارد.

هشت هفته تمرینات عصبی-عضلانی با و بدون مداخلات شناختی بر فعالیت الکترومایوگرافی ورزشکاران زن مستعد آسیب ACL تأثیر دارد.

روش انجام پژوهش

طراحی مطالعه

کارآزمایی کنترل شده تصادفی

معیارهای ورود

شرکت‌کنندگان واجد شرایط برای ورود به مطالعه باید تمامی شرایط زیر را دارا می‌بودند:

- بازیکن زن والیبال با محدوده سنی ۱۸ تا ۲۳ سال؛
- سابقه حداقل سه سال فعالیت ورزشی در سطح رقابتی؛
- وجود والگوس داینامیک زانو تأییدشده از طریق آزمون‌های غربالگری، شامل:
  - مشاهده والگوس داینامیک زانو در آزمون پرش تاک همراه با تکلیف شناختی؛
  - زاویه والگوس داینامیک زانو بیش از ۱۲ درجه در تکلیف فرود تک‌پا همراه با تکلیف شناختی؛
- نداشتن آسیب اندام تحتانی در شش ماه گذشته که نیازمند درمان پزشکی یا توقف فعالیت ورزشی بوده باشد؛
- نداشتن سابقه جراحی زانو یا مشکلات سیستم عصبی-مرکزی؛
- توانایی و تمایل به شرکت در جلسات تمرینی سه بار در هفته به مدت هشت هفته متوالی؛
- امکان حضور در جلسات ارزیابی پیش‌آزمون و پس‌آزمون.

معیارهای خروج

شرکت‌کنندگانی که هر یک از شرایط زیر را داشتند از مطالعه کنار گذاشته شدند:

- وجود آسیب یا درد اندام تحتانی که مانع از مشارکت در فعالیت‌های ورزشی شود؛
- غیبت در دو جلسه تمرینی متوالی یا سه جلسه تمرینی غیرمتوالی از برنامه مداخله؛
- احساس درد یا بروز آسیب حین اجرای پروتکل تمرینی.

روش نمونه‌گیری و حجم نمونه

جامعه آماری این پژوهش شامل بازیکنان والیبال زن باشگاهی و دانشگاهی شهر همدان خواهد بود. شرکت‌کنندگان از طریق شناسایی تیم‌های فعال در سطح باشگاهی و دانشگاهی و با هماهنگی مربیان و سرپرستان تیم‌ها دعوت خواهند شد.

برای تعیین حجم نمونه، از نرم‌افزار GPower نسخه ۳/۱ و آزمون Repeated Measures ANOVA within; between interaction استفاده خواهد شد. با در نظر گرفتن سطح معناداری ۰/۰۵، توان آماری ۰/۸۵ و اندازه اثر ۰/۳۱ (بر اساس داده‌های زاویه DKV در مطالعه Hamoongard et al., 2022)، حداقل حجم نمونه موردنیاز ۲۶ نفر برآورد خواهد شد. با توجه به پیش‌بینی ریزش ۱۵ درصدی، حجم نمونه به ۳۰ نفر افزایش داده خواهد شد. در نهایت، ۱۵ ورزشکار زن دارای DKV در هر یک از دو گروه تمرین و کنترل تخصیص داده خواهند شد.

روند پژوهش

به منظور حصول اطمینان از رضایت افراد برای شرکت در این پژوهش، فرم رضایت نامه ی کتبی در اختیار آنان قرار خواهد گرفت و تمام خطرات احتمالی آزمون برای آزمودنی شرح داده خواهد شد. افراد شرکت کننده در آزمون برای شرکت یا عدم شرکت در آزمون آزاد هستند و پس از اعلام موافقت خود نیز، هرزمانی که بخواهند می توانند از آزمون خارج شوند. خروج از پژوهش باعث محرومیت آزمودنی ها از دریافت خدمات مراقبتی ، تشخیصی و درمانی معمول نخواهد شد و مستلزم پرداخت جریمه نخواهند بود. هیچ گونه هزینه ای جهت شرکت در این تحقیق بر عهده آزمودنی نیست و تمامی اطلاعاتی که جهت شرکت در آزمون از آزمودنی گرفته خواهد شد محرمانه خواهد ماند. پس از ثبت نام، ورزشکاران در دو گروه تجربی و کنترل طبقه بندی می شوند. گروه تمرینی به مدت هشت هفته عصبی-عضلانی-شناختی مورد نظر را دریافت میکنند درحالیکه گروه کنترل در این هشت هفته تنها تمرینات عصبی-عضلانی دریافت خواهند کرد. با استفاده از نرم افزار Random Number Generator، شرکت کنندگان به طور تصادفی در یکی از گروه ها قرار خواهند گرفت. پنهان سازی تخصیص با تکنیک پاکت های مهر و موم شده مات با شماره ترتیبی انجام می شود.

مداخله اصلاحی

مداخله طراحی‌شده از دو مؤلفه اصلی تشکیل خواهد شد: تمرینات عصبی-عضلانی و تمرینات شناختی. تمرینات عصبی-عضلانی با هدف بهبود تعادل پویا، افزایش قدرت عضلات کلیدی اندام تحتانی (به‌ویژه ابداکتورهای لگن، چهارسر ران و همسترینگ)، ارتقای حس عمقی و اصلاح الگوهای حرکتی پرخطر طراحی خواهند شد. این تمرینات شامل حرکات چندصفحه‌ای، تمرینات تعادلی و تمرینات پرش و فرود در شرایط مختلف خواهند بود.

تمرینات شناختی به‌صورت یکپارچه در دل تمرینات حرکتی گنجانده خواهند شد و هدف آن‌ها ایجاد شرایط دوگانه‌ای خواهد بود که در آن ورزشکاران هم‌زمان با اجرای حرکات درگیر پردازش‌های شناختی شوند. این تمرینات چهار حوزه کلیدی را هدف قرار خواهند داد:

۱) تصمیم‌گیری سریع (پاسخ به محرک‌های دیداری و شنیداری)،

۲) حافظه کاری (به‌خاطر سپردن و اجرای توالی‌های حرکتی یا عددی)،

۳) مهار پاسخ (اجتناب از واکنش به محرک‌های نامربوط)،

۴) تقسیم توجه (تمرکز هم‌زمان بر توپ یا هم‌تیمی و اجرای صحیح حرکت).

تمرینات شناختی به‌گونه‌ای طراحی خواهند شد که ساده، کاربردی، تدریجی و مرتبط با نیازهای عملکردی ورزش والیبال باشند و به‌صورت زوجی اجرا خواهند شد تا بازخورد همتایان نیز در بهبود عملکرد مؤثر باشد.

ساختار کلی برنامه تمرینی

برنامه تمرینی در قالب یک مداخله هشت‌هفته‌ای شامل سه جلسه در هفته و در مجموع ۲۴ جلسه اجرا خواهد شد. هر جلسه ۶۰ دقیقه و در روزهای غیرمتوالی برگزار خواهد شد تا فرصت کافی برای ریکاوری فراهم شود. جلسات در زمان‌بندی تمرینات عادی تیم‌ها ادغام خواهند شد تا از تداخل با برنامه ورزشی جلوگیری شود و پایبندی افزایش یابد.

ساختار هر جلسه شامل سه بخش خواهد بود:

گرم‌کردن (۱۵ دقیقه): حرکات کششی پویا، فعالیت هوازی سبک، حرکات اختصاصی والیبال

تمرین اصلی (۴۰-۳۰ دقیقه): تمرینات عصبی—عضلانی و شناختی با بار تدریجی

سردکردن (۱۰ دقیقه): کشش ایستا و تمرینات تنفسی

فازهای تمرینی

پروتکل تمرینی در چهار فاز متوالی و پیش‌رونده طراحی خواهد شد که هر فاز دو هفته به طول خواهد انجامید:

فاز اول: کنترل حرکت (هفته‌های ۱ و ۲)

تمرکز بر اصلاح الگوهای حرکتی در صفحه ساجیتال و کاهش والگوس داینامیک زانو خواهد بود.

تمرینات ساده، دوپا، با بازخورد دیداری و کلامی زیاد، روی سطوح پایدار و با شدت کم تا متوسط اجرا خواهند شد.

فاز دوم: برنامه‌ریزی راهبردی (هفته‌های ۳ و ۴)

بار شناختی افزایش خواهد یافت.

تمرینات تک‌پا، تصمیم‌گیری، واکنش به محرک‌های دیداری و شنیداری اضافه خواهند شد.

بازخورد خارجی کاهش خواهد یافت تا اتکای فرد به حس عمقی افزایش یابد.

فاز سوم: پردازش خودکار (هفته‌های ۵ و ۶)

هدف، خودکارسازی الگوهای حرکتی در شرایط دوگانه خواهد بود.

تمرینات شامل حرکات چندجهتی، تک‌پا، با بار شناختی بالا و سرعت و شدت بیشتر خواهند بود.

فاز چهارم: انتقال و تثبیت (هفته‌های ۷ و ۸)

هدف انتقال مهارت‌ها به شرایط واقعی مسابقه خواهد بود.

تمرینات شامل حرکات تک‌پا در صفحه فرونتال، محرک‌های غیرقابل‌پیش‌بینی و سناریوهای خاص والیبال (مانند اسلاید دفاعی و تغییر جهت سریع) خواهند بود.

تمرینات با شدت بالا، تحت خستگی و فشار زمانی اجرا خواهند شد.

معیارهای پیشرفت بین فازها

پیشرفت آزمودنی‌ها از یک فاز به فاز بعد تنها در صورتی مجاز خواهد بود که فرد در سه جلسه متوالی، معیارهای عملکردی از پیش تعیین‌شده را کسب کند. این معیارها شامل:

بخش حرکتی: ارزیابی راستای زانو و کیفیت اجرای حرکات توسط مربی آموزش‌دیده

بخش شناختی: تعداد خطاها، حفظ کیفیت حرکت در شرایط دوگانه، سرعت و دقت پاسخ‌دهی

در صورت عدم کسب معیارها، آزمودنی در همان فاز باقی خواهد ماند و تمرینات جایگزین با سطح دشواری مشابه برای او در نظر گرفته خواهد شد. تمامی تصمیمات در فرم‌های استاندارد ثبت و هفتگی توسط متخصص ورزشی بازبینی خواهند شد.

مداخله گروه کنترل

گروه کنترل در یک مداخله فعال شرکت خواهد کرد که شامل همان تمرینات عصبی–عضلانی گروه تجربی خواهد بود، با این تفاوت که تمرینات فقط در شرایط تک‌وظیفه‌ای و بدون مؤلفه شناختی اجرا خواهند شد. ساختار جلسات، تعداد، مدت و شدت تمرینات در هر دو گروه یکسان خواهد بود تا اثر حجم تمرین، توجه مربی و تعامل اجتماعی کنترل شود.

تمرینات گروه کنترل نیز در چهار فاز پیش‌رونده اجرا خواهند شد، اما بدون بار شناختی. راهبردهای آموزشی شامل دستورالعمل کلامی، نمایش تمرین و بازخورد اصلاحی خواهند بود و تمرکز صرفاً بر اجرای فیزیکی تمرینات خواهد بود. این طراحی امکان بررسی اثر خالص مؤلفه شناختی را فراهم خواهد کرد.

روش آماری

تحلیل‌های آماری با استفاده از نرم‌افزار SPSS نسخه ۲۷ انجام خواهد شد. آمار توصیفی شامل میانگین و انحراف معیار برای متغیرهای پیوسته گزارش خواهد شد. آزمون شاپیرو–ویلک برای بررسی نرمال‌بودن توزیع داده‌ها به‌کار گرفته خواهد شد. برای مقایسه متغیرهای جمعیت‌شناختی بین گروه‌ها از آزمون تی مستقل استفاده خواهد شد.

برای بررسی اثرات متقابل گروه (تمرین/کنترل) و زمان (پیش‌آزمون/پس‌آزمون) از تحلیل واریانس با اندازه‌گیری مکرر (Repeated Measures ANOVA) استفاده خواهد شد. مقایسه‌های زوجی با اصلاح بونفرونی جهت کنترل خطای نوع اول انجام خواهد شد. اندازه اثر با استفاده از شاخص Partial Eta Squared محاسبه خواهد شد.

برای ارزیابی معناداری بالینی و اثربخشی مداخله، حداقل تغییر معنادار بالینی (MCID) و حداقل تغییر قابل‌تشخیص (MDC) در سطح اطمینان ۹۵ درصد محاسبه خواهند شد. مقدار MDC با استفاده از خطای استاندارد اندازه‌گیری (SEM) و فرمول زیر محاسبه خواهد شد:

$$\mathrm{SEM} = \mathrm{SD}_{pre} \times \sqrt{(1 - \mathrm{ICC})}$$

$$\mathrm{MDC}_{\%95} = 1.96 \times \sqrt{2} \times \mathrm{SEM}$$

همچنین برای تعیین MCID از شاخص Reliable Change Index (RCI) استفاده خواهد شد:

$$\mathrm{MCID}_{RCI} = 1.96 \times \mathrm{SD}_{pre}\ [\sqrt{(2 \times (1 - \mathrm{ICC}))}]$$

در این فرمول‌ها،

SDpre نشان‌دهنده انحراف معیار مقادیر پیش‌آزمون در گروه تمرین و

ICC ضریب همبستگی درون‌کلاسی (شاخص پایایی آزمون–بازآزمون) خواهد بود.

منابع

۱. Cowling EJ, Steele JR. Is lower limb muscle synchrony during landing affected by gender? Implications for variations in ACL injury rates. Journal of Electromyography and Kinesiology. 2001;11(4):263-268.

۲. Levangie PK, Norkin CC. Joint structure and function: a comprehensive analysis: FA Davis 2011.

۳. Smith TO, Davies L, Hing CB. Early versus delayed surgery for anterior cruciate ligament reconstruction: a systematic review and meta-analysis. Knee surgery, sports traumatology, arthroscopy. 2010;18:304-۳۱۱.

۴. Hewett TE, Ford KR, Hoogenboom BJ, et al. Understanding and preventing acl injuries: current biomechanical and epidemiologic considerations-update 2010. North American journal of sports physical therapy: NAJSPT. 2010;5(4):234.

۵. Imwalle LE, Myer GD, Ford KR, et al. Relationship between hip and knee kinematics in athletic women during cutting maneuvers: a possible link to noncontact anterior cruciate ligament injury and prevention. Journal of strength and conditioning research/National Strength & Conditioning Association. 2009;23(8):2223.

۶. Quatman CE, Hewett TE. The anterior cruciate ligament injury controversy: is “valgus collapse” a sex-specific mechanism? British journal of sports medicine. 2009;43(5):328-335.

.۷ Giesche F, Wilke J, Engeroff T, et al. Are biomechanical stability deficits during unplanned single-leg landings related to specific markers of cognitive function? Journal of science and medicine in sport. 2020;23(1):82-88.

.۸ DiStefano LJ, Root HJ, Frank BS, et al. Implementation strategies for ACL injury prevention programs. ACL injuries in the female athlete: causes, impacts, and conditioning programs. 2018:625-639.

.۹ Alentorn-Geli E, Myer GD, Silvers HJ, et al. Prevention of non-contact anterior cruciate ligament injuries in soccer players. Part 1: Mechanisms of injury and underlying risk factors. Knee surgery, sports traumatology, arthroscopy. 2009;17:705-729.

.۱۰ Hart JM, Grindstaff TL, Beazell JR, et al. Assessment of ankle dorsiflexion range of motion restriction. Athletic Training & Sports Health Care. 2009;1(1):7-8.

.۱۱ Herrington L, Myer GD, Munro A. Intra and inter-tester reliability of the tuck jump assessment. Physical Therapy in Sport. 2013;14(3):152-155.

.۱۲ Hewett TE, Myer GD, Ford KR, et al. Biomechanical measures of neuromuscular control and valgus loading of the knee predict anterior cruciate ligament injury risk in female athletes: a prospective study. The American journal of sports medicine. 2005;33(4):492-501.

.۱۳ Grooms DR, Onate JA. Neuroscience application to noncontact anterior cruciate ligament injury prevention. Sports Health. 2016;8(2):149-152.

.۱۴ Bonnette S, Diekfuss JA, Grooms DR, et al. Electrocortical dynamics differentiate athletes exhibiting low-and high-ACL injury risk biomechanics. Psychophysiology. 2020;57(4):e13530.

.۱۵ Diekfuss JA, Grooms DR, Yuan W, et al. Does brain functional connectivity contribute to musculoskeletal injury? A preliminary prospective analysis of a neural biomarker of ACL injury risk. Journal of science and medicine in sport. 2019;22(2):169-174.

.۱۶ Mandelbaum BR, Silvers HJ, Watanabe DS, et al. Effectiveness of a neuromuscular and proprioceptive training program in preventing anterior cruciate ligament injuries in female athletes: 2-year follow-up. The American journal of sports medicine. 2005;33(7):1003-1010.

.۱۷ Nagai T, Schilaty ND, Strauss JD, et al. Analysis of lower extremity proprioception for anterior cruciate ligament injury prevention: current opinion. Sports Medicine. 2018;48(6):1303-1309.

.۱۸ Liu R, Liu J, Ma X, et al. Effect of FIFA 11+ intervention on change of direction performance in soccer and futsal players: A systematic review and meta-analysis. International Journal of Sports Science & Coaching. 2021;16(3):862-872.

.۱۹ Junge A, Dvorak J. Injury risk of playing football in Futsal World Cups. British journal of sports medicine. 2010;44(15):1089-1092.

.۲۰ Dai B, Cook RF, Meyer EA, et al. The effect of a secondary cognitive task on landing mechanics and jump performance. Sports biomechanics. 2018;17(2):1.۲۰۵-۹۲

.۲۱ Nazary-Moghadam S, Salavati M, Esteki A, et al. Reliability of kinematic measures in subjects with anterior cruciate ligament deficiency during dual-task walking. Journal of bodywork and movement therapies. 2017;21(4):852-859.

.۲۲ Almonroeder TG, Kernozek T, Cobb S, et al. Divided attention during cutting influences lower extremity mechanics in female athletes. Sports biomechanics. 2019;18(3):264-276.

.۲۳ Monfort SM, Pradarelli JJ, Grooms DR, et al. Visual-spatial memory deficits are related to increased knee valgus angle during a sport-specific sidestep cut. The American journal of sports medicine. 2019;47(6):1488-1495.

.۲۴ Pashler H. Dual-task interference in simple tasks: data and theory. Psychological bulletin. 1994;116(2):220.

.۲۵ Ruthruff E, Van Selst M, Johnston JC, et al. How does practice reduce dual-task interference: Integration, automatization, or just stage-shortening? Psychological research. 2006;70:125-142.
